# Beyond the Hippocampus and the SVZ: Adult Neurogenesis Throughout the Brain

**DOI:** 10.3389/fncel.2020.576444

**Published:** 2020-09-29

**Authors:** Michal P. Jurkowski, Luis Bettio, Emma K. Woo, Anna Patten, Suk-Yu Yau, Joana Gil-Mohapel

**Affiliations:** ^1^Island Medical Program, University of British Columbia, Vancouver, BC, Canada; ^2^Division of Medical Sciences, University of Victoria, Victoria, BC, Canada; ^3^Centre for Interprofessional Clinical Simulation Learning (CICSL), Royal Jubilee Hospital, Victoria, BC, Canada; ^4^Department of Rehabilitation Sciences, Hong Kong Polytechnic University, Hung Hom, Hong Kong

**Keywords:** adult neurogenesis, amygdala, cortex, hippocampus, hypothalamus, striatum, substantia nigra, subventricular zone

## Abstract

Convincing evidence has repeatedly shown that new neurons are produced in the mammalian brain into adulthood. Adult neurogenesis has been best described in the hippocampus and the subventricular zone (SVZ), in which a series of distinct stages of neuronal development has been well characterized. However, more recently, new neurons have also been found in other brain regions of the adult mammalian brain, including the hypothalamus, striatum, substantia nigra, cortex, and amygdala. While some studies have suggested that these new neurons originate from endogenous stem cell pools located within these brain regions, others have shown the migration of neurons from the SVZ to these regions. Notably, it has been shown that the generation of new neurons in these brain regions is impacted by neurologic processes such as stroke/ischemia and neurodegenerative disorders. Furthermore, numerous factors such as neurotrophic support, pharmacologic interventions, environmental exposures, and stem cell therapy can modulate this endogenous process. While the presence and significance of adult neurogenesis in the human brain (and particularly outside of the classical neurogenic regions) is still an area of debate, this intrinsic neurogenic potential and its possible regulation through therapeutic measures present an exciting alternative for the treatment of several neurologic conditions. This review summarizes evidence in support of the classic and novel neurogenic zones present within the mammalian brain and discusses the functional significance of these new neurons as well as the factors that regulate their production. Finally, it also discusses the potential clinical applications of promoting neurogenesis outside of the classical neurogenic niches, particularly in the hypothalamus, cortex, striatum, substantia nigra, and amygdala.

## Introduction

Over the past 50 years, it has become increasingly evident that the adult mammalian brain retains the capacity to generate new neurons (Altman, 1962; Altman and Das, 1965; Kaplan and Hinds, 1977; Cameron et al., 1993; Kuhn et al., 1996) and that this characteristic is preserved in humans (Eriksson et al., 1998; Bergmann et al., 2015; Boldrini et al., 2018). However, this process is not ubiquitous. Indeed cell proliferation and neuronal differentiation only continue to occur throughout the lifespan of an individual in specific and restricted areas of the brain. The hippocampus (Bonaguidi et al., 2012; Kempermann et al., 2015) and the subventricular zone (SVZ; Doetsch et al., 1997, 1999; García-Verdugo et al., 1998; Ponti et al., 2017) are the two most-studied neurogenic niches in which adult neurogenesis has been extensively described and where several well-characterized stages of the neurogenic process have been defined.

**Figure 1:**
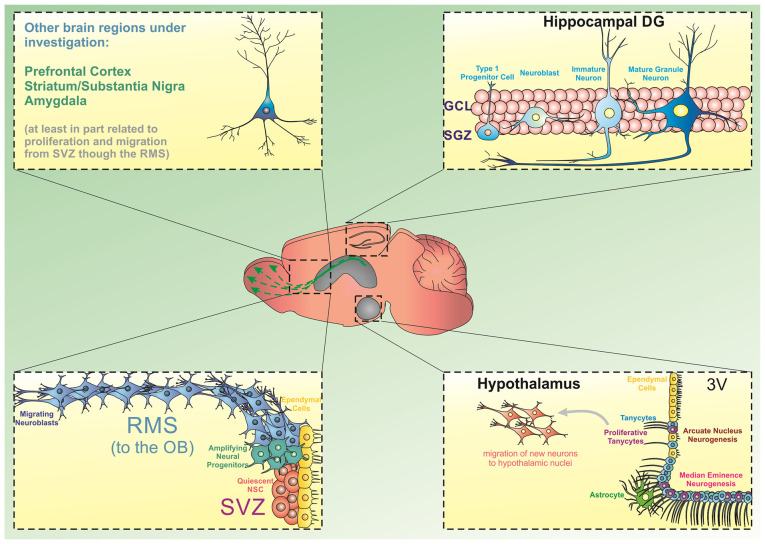
Source of progenitor cells in different brain regions. The generation of new neurons from stem/progenitor cells has been extensively described in the hippocampal dentate gyrus (DG) and the subventricular zone/olfactory bulb (SVZ/OB). In the DG, precursor cells located in the subgranular zone divide and give rise to amplifying cells, which can commit to a neuronal phenotype and move into the granule cell layer to integrate into existing hippocampal circuitries. Similarly, dividing progenitor cells in the SVZ can differentiate into neural progenitors and migrate through the rostral migratory stream (RMS) towards the OB. Besides these two regions, there is emerging evidence indicating that the hypothalamic arcuate nucleus and the median eminence present neurogenic capacity. Particularly, a subpopulation of tanycytes has been shown to display neurogenic characteristics in these subregions. Additionally, experimental evidence has suggested that progenitor cells can deviate from the RMS and differentiate and mature into other brain regions including the prefrontal cortex, striatum, substantia nigra, and amygdala.

However, several additional areas of the brain have emerged as containing newly generated neurons beyond early development. Animal studies have shown that these neurogenic areas include the hypothalamus (Evans et al., 2002), striatum (Parent et al., 1995; Suzuki and Goldman, 2003; Shapiro et al., 2009), substantia nigra (SN; Cassidy et al., 2003), cortex (Magavi et al., 2000), and amygdala (Bernier et al., 2002). Some evidences show that the new neurons in these novel neurogenic areas arise from migrating neural stem and progenitor cells (NSPCs), typically originating in the SVZ (Bernier et al., 2002; Cao et al., 2002; Dayer et al., 2005; Inta et al., 2008; Shapiro et al., 2009; Huttner et al., 2014). Other studies show that endogenous pools of NSPCs may actually exist within these regions, allowing them to replicate and populate local neuronal circuits (Parent et al., 1995; Zecevic and Rakic, 2001; Evans et al., 2002; Jhaveri et al., 2018; Figure 1).

**Figure 2:**
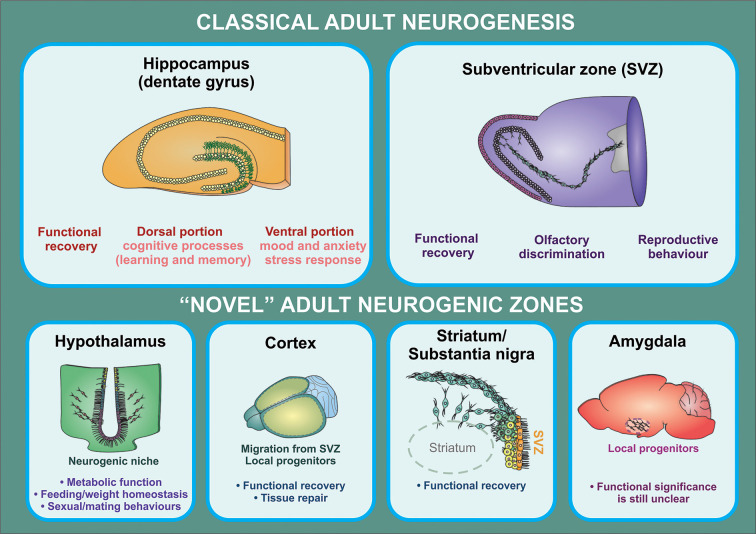
Functional implications of adult neurogenesis. The contribution of adult neurogenesis to physiological and pathological processes mediated by the hippocampus and the OB is supported by a substantial amount of evidence from rodent studies. In the hippocampus, this process regulates specific aspects of cognitive (dorsal portion) and affective (ventral portion) processing, while newborn neurons generated in the SVZ were shown to contribute to olfactory function and reproductive behavior (in birds). There is also increasing evidence indicating that neural progenitors may exert a relevant role in the regulation of hypothalamic function (particularly in metabolic function/feeding behaviors). On the other hand, it is still unclear whether progenitor cells found in other brain regions have a functional significance other than participating in recovery processes triggered following injury, neuronal loss, or neurodegeneration.

Generation of new neurons in these novel neurogenic areas may serve important functional roles. Neurogenesis in the hypothalamus has the potential to affect metabolism and fat storage, as shown in multiple high-fat diet (HFD) studies in mice (Kokoeva, 2005; Lee et al., 2014). Neurogenesis in the hypothalamus may also play a role in behavioral and sexual function (Bernstein et al., 1993; Fowler et al., 2002; Cheng et al., 2004). In the amygdala, neurogenesis may play a role in fear conditioning and stress response (Shapiro et al., 2009; Saul et al., 2015). The functional significance of new neurons is less well characterized in the striatum, SN, and cortex (Figure 2). That said, the importance of endogenous neurogenesis in the context of disease mechanisms that affect these brain regions cannot be understated (Kay and Blum, 2000; Mohapel et al., 2005; Huttner et al., 2014; Moraga et al., 2014). Further investigation of these neurogenic zones also shows that their proliferation can be altered by growth factors (Pencea et al., 2001b; Yoshikawa et al., 2010; Zhu et al., 2011), pharmacologic treatments (Rojczyk et al., 2015), and environmental exposures (Kisliouk et al., 2014; Niwa et al., 2016). These findings offer an exciting possibility for the management of neurologic diseases in the future.

Though the existence of neurogenesis in the adult human brain remains an ongoing area of debate, recent advances have allowed us to investigate whether the findings from animal studies correlate with what occurs in the adult human brain (Spalding et al., 2013; Mathews et al., 2017; Boldrini et al., 2018; Sorrells et al., 2018). Here we provide an overview of the evidence for neurogenesis in brain regions beyond the hippocampus and the SVZ, its functional significance and modifying factors as well as its potential relevance in the context of acute and chronic neurologic diseases.

## “Classical” Adult Neurogenic Zones

The two brain regions where the process of neurogenesis has been best described are the hippocampus and the subventricular zone/olfactory bulb (SVZ/OB; Figure 1). In both the hippocampus and the SVZ/OB, the neurogenic process can be subdivided into well-defined stages, from cell proliferation to neuronal differentiation, maturation, and functional (i.e., synaptic) integration. In the hippocampus, adult neurogenesis is thought to play a role in both mood regulation (i.e., affective behaviors) and cognition (i.e., learning, memory, and spatial navigation). Similarly, in the SVZ/OB, adult neurogenesis is thought to contribute to optimal olfactory circuit formation.

### Neurogenesis in the Hippocampus

The hippocampus is part of the limbic system and, in humans, is located deeply within the medial temporal lobe. The hippocampus is arguably one of the most plastic regions of the brain, showing not only the capacity to undergo neurogenesis but also other types of structural and functional (i.e., synaptic) plasticity. These properties are integral to the function of the hippocampus and its role in mood regulation and cognition (namely, learning, and memory). During the process of adult hippocampal neurogenesis, newborn neurons migrate just a short distance from the dentate gyrus (DG) subgranular zone (SGZ) to the DG granule zone, where they integrate into the existing neuronal circuitry.

#### Stages of Hippocampal Neurogenesis

Hippocampal neurogenesis is a highly regulated process that involves four distinct phases: (1) the precursor cell phase; (2) the early survival phase; (3) the post-mitotic phase; and (4) the late survival phase (Kempermann et al., 2004, 2015; Bonaguidi et al., 2012). During the precursor cell phase, type-1 cells divide asymmetrically, giving rise to transit amplifying cells (type-2 cells). Type-1 cells are radial-glia-like cells with triangular somas and branches projecting into the inner molecular layer that express the undifferentiated neural progenitor cell (NPC) marker nestin as well as glial fibrillary acidic protein (GFAP), one of several astrocytic features that they demonstrate (Seri et al., 2001; Filippov et al., 2003). Type-2 cells are short and wide. They have a dense, irregular-shaped nucleus and are GFAP-negative. These transient cells are capable of tangential migration and are highly proliferative. Type-2 cells can be further characterized based on the expression of the immature neuronal marker doublecortin (DCX) into either DCX-negative type-2A cells (less differentiated) and DCX-expressing type-2B cells (more differentiated and committed to the neuronal lineage; Brown et al., 2003; Filippov et al., 2003; Kronenberg et al., 2003). Type-2B cells further differentiate into type-3 cells, which are DCX-positive and nestin-negative and are morphologically distinct from type-2 cells because of their round nucleus (Brandt et al., 2003). Type-3 cells also express the neuronal marker polysialylated neuronal cell adhesion molecule (PSA-NCAM; Seki, 2002). Type-1, type-2A, type-2B, and type-3 cells together comprise the spectrum of precursor cells in the hippocampal SGZ, with type-2A, type-2B, and type-3 cells accounting for the majority of proliferation that occurs within this region (Kempermann et al., 2015). Within 3 days of cell division, this cell population can increase four- to fivefold, and newly generated cells enter a post-mitotic stage characterized by the expression of post-mitotic neuronal markers—neuronal nuclei (NeuN) and calretinin (CR; Brandt et al., 2003; Kempermann et al., 2003). The number of immature neurons (neuroblasts) rapidly declines over the subsequent 4 days due to widespread apoptosis and then stabilizes at around 4 weeks, at which point approximately 20% of the newly generated neurons have survived and incorporated into the existing neuronal circuitry (Biebl et al., 2000; Kempermann et al., 2003; Kuhn et al., 2005). Indeed a few days after exiting the cell cycle, newly generated neuroblasts begin attempting to establish functional connections in the target hippocampal cornu ammonis (CA) 3 region. Immature neurons that are able to establish functional connections will then receive neurotransmitter signals as well as trophic support from pro-survival factors (Tashiro et al., 2006; Imielski et al., 2012; Cancino et al., 2013; Ramirez-Rodriguez et al., 2013). Notably, at this stage, immature neurons receive primarily gamma-aminobutyric acid (GABA)ergic input and transiently express a Na^+^/K^+^/Cl^−^ co-transporter that actively transports Cl^−^ against its concentration gradient and into the cell. As a consequence, GABA signaling has an overall excitatory effect by allowing Cl^−^ to move according to its concentration gradient (i.e., out of the cell) and depolarizing the intracellular space, a process thought to contribute to neuronal maturation (Rivera et al., 1999; Ganguly et al., 2001; Ben-Ari, 2002; Ge et al., 2006). Following this stage, the final cell number is relatively established, and only a small number of cells are eliminated during the maturation stage (Kempermann et al., 2003). Granule cells then begin their functional maturation, transitioning to normal membrane properties involving physical changes of size, length, thickness, and branching of dendrites and electrochemical changes including increased membrane capacitance and reduced membrane resistance (van Praag et al., 2002; Marin-Burgin et al., 2012). Maturing granule cells eventually develop glutamatergic connections and become electrophysiologically identical to the existing neurons (Wang et al., 2000; van Praag et al., 2002; Ambrogini et al., 2004; Schmidt-Hieber et al., 2004), thus completing functional integration into the existing hippocampal circuitry.

#### Regulation of Hippocampal Neurogenesis

Maintenance of an endogenous pool of type-1 cells is key to the preservation of hippocampal neurogenesis throughout adulthood, and this process is therefore regulated by a number of factors. The transcription factor sex-determining region Y-box 2 (Sox 2) appears to play a central role in this process by regulating several intracellular signaling pathways (Steiner et al., 2006). First, Sox 2 controls the expression of Sonic hedgehog (Shh), which in turn promotes the proliferation of type-1 cells (Favaro et al., 2009). In addition, Sox 2 inhibits Wnt signaling, thereby maintaining the cells in a proliferative state (Kuwabara et al., 2009).

Following cell proliferation, brain-derived neurotrophic factor (BDNF) plays an important role in the early cell survival phase. BDNF activates the tropomyosin receptor kinase B (TrKB) receptor, which acts *via* protein kinase C to activate proteins involved in cell survival and cell migration (Ortiz-López et al., 2017). In addition to regulating hippocampal cell proliferation, Wnt signaling is also involved in neuronal cell differentiation by regulating the expression of the transcription factors neuronal differentiation 1 (NeuroD1) and prospero-related homeobox 1 (Kuwabara et al., 2009; Gao et al., 2011; Karalay et al., 2011). cAMP-response element-binding (CREB) protein is another important factor in neuronal maturation. Similar to NeuroD1, CREB enhances neurite outgrowth and dendritic branching while being positively regulated by GABAergic signaling (Fujioka, 2004; Tozuka et al., 2005; Gao et al., 2009; Jagasia et al., 2009). The numerous factors involved in lineage progression are connected through complex cross-talk signaling pathways, such that if one factor is impaired, the entire neurogenic cycle is halted (Zhang C. L. et al., 2006; Niu et al., 2011; Shimozaki et al., 2013).

In addition to transcription factors and signaling pathways, adult hippocampal neurogenesis can also be modulated by various intrinsic and extrinsic factors such as the activation of the hypothalamus–pituitary–adrenal (HPA) axis (Schloesser et al., 2009; Snyder et al., 2011), which leads to elevated blood levels of glucocorticoids (McEwen et al., 1992; Anacker et al., 2013) in response to chronic stress exposure (Gould et al., 1998; Murray et al., 2008). Thus, aberrant stress responses inherent in a variety of psychiatric conditions can downregulate adult neurogenesis. Other factors that have been shown to possess a negative effect on adult hippocampal neurogenesis include pro-inflammatory factors (Ekdahl et al., 2003), angiotensin II receptor antagonists (Mukuda and Sugiyama, 2007), testosterone at specific times during the lifespan (Allen et al., 2014, 2015; Zhang et al., 2014), and aging (Kuhn et al., 1996; Ben Abdallah et al., 2010; Gil-Mohapel et al., 2013).

Conversely, selective serotonin reuptake inhibitors (Malberg et al., 2000; Santarelli et al., 2003; Banasr et al., 2006; Surget et al., 2008, 2011) as well as several non-pharmacologic interventions including electroconvulsive therapy (Zilles et al., 2015; Olesen et al., 2017; Wang et al., 2017), environmental enrichment (Kempermann et al., 1997; Gualtieri et al., 2017), caloric restriction (Lee et al., 2002; Stangl and Thuret, 2009), and physical exercise (Van Praag et al., 1999; Yau et al., 2011, 2012; Yau S.-Y. et al., 2014; Yau S. et al., 2014; Firth et al., 2018; Nguemeni et al., 2018) have all been repeatedly shown to potentiate adult hippocampal neurogenesis. Estrogen (Brännvall et al., 2002; Perez-Martin et al., 2003) and angiotensin II (Mukuda et al., 2014) also have the capacity to stimulate the endogenous neurogenic process in the hippocampus.

Notably, some of these strategies, including physical exercise and environmental enrichment, have also been shown to improve degenerative changes associated with various neurodegenerative conditions such as Alzheimer’s disease (Paillard et al., 2015; Vivar, 2015; Ryan and Kelly, 2016), Parkinson’s disease (PD; Ang et al., 2010; Lamm et al., 2014; Paillard et al., 2015; Vivar, 2015), and Huntington’s disease (HD; Vivar, 2015). Although the exact mechanisms that underlie the beneficial effects of physical exercise and environmental enrichment are not completely understood, a reduction in adult hippocampal neurogenesis has been observed in several animal models of these neurodegenerative disorders (Ang et al., 2010; Lamm et al., 2014; Paillard et al., 2015; Vivar, 2015; Ryan and Kelly, 2016). Thus, it is likely that an increase in hippocampal neurogenic capacity might contribute to these beneficial effects.

#### Functions of Hippocampal Neurogenesis

The ventral portion of the hippocampus is thought to be particularly involved in mood regulation and affective behaviors through its connections to the amygdala, nucleus accumbens, and hypothalamus (Anacker et al., 2013). Indeed chronic stress has been shown to preferentially affect the ventral hippocampus (Mirescu et al., 2004). As previously mentioned, the effects of stress are primarily mediated through the HPA axis and subsequent glucocorticoid production. Increased corticosterone levels alone appear to be sufficient to induce depressive-like and anxiety-like behaviors in rodents (Schloesser et al., 2009; Snyder et al., 2011). Conversely, several studies have also shown that damage to the ventral hippocampus is associated with an increase in anxiety-like (Bergami et al., 2008; Revest et al., 2009) and depression-like (Santarelli et al., 2003; Jiang et al., 2005; Airan et al., 2007) behaviors.

The dorsal hippocampus is functionally involved in certain aspects of cognition (namely, spatial navigation, learning, and memory) through connections with various cortical areas (Anacker and Hen, 2017). As mentioned above, the central role that the hippocampus plays in these aspects of cognition is due to its ability to undergo both structural and functional (i.e., synaptic) plasticity in response to stimuli. Notably, newly generated hippocampal neurons are particularly receptive to behavior-dependent synaptic plasticity. They receive input from other neurons (Bruel-Jungerman et al., 2006; Toni et al., 2007) and have a low threshold for long-term potentiation (Snyder et al., 2001). In agreement, several studies have shown that new neurons are recruited into hippocampal neuronal circuits in response to spatial learning (Gould et al., 1999a; Kee et al., 2007), while others have shown that certain aspects of spatial learning can be impaired through ablation of adult hippocampal neurogenesis (Jessberger et al., 2009) and with age (where spatially imprecise navigation strategies are used due to a decline in neurogenic capacity; Gil-Mohapel et al., 2013).

There is also empirical evidence suggesting that the dorsal DG plays a role in pattern separation (whereas the CA3 region appears to be involved in pattern completion; Clelland et al., 2009; Deng et al., 2010; Aimone et al., 2011; Nakashiba et al., 2012; Hunsaker and Kesner, 2013), and studies have shown an involvement of the hippocampus in both spatial (Clelland et al., 2009; Creer et al., 2010; Tronel et al., 2012; Déry et al., 2013) and temporal pattern separation (Koehl and Abrous, 2011). This is further supported by the finding that adult DG neurogenesis plays an important role in preventing memory interference (Garthe et al., 2009). Notably, neurogenesis in the dorsal hippocampus has also been shown to play a role in the consolidation and the reorganization of explicit memories (Kitamura et al., 2009).

The relevance of adult neurogenesis for hippocampal function in the primate brain has been intensively debated, and recent evidence suggests that this process probably plays a role in human cognition. For instance, a correlation between cognitive performance and neurogenic markers was observed in the monkey DG, as well as an age-related decline in proliferation/maturation markers (Ngwenya et al., 2015). Similarly, the presence of neuronal progenitors and immature neurons was recently reported in the human DG during physiological aging, and the number of these cells was found to be drastically affected by pathological conditions (Moreno-Jiménez et al., 2019; Seki et al., 2019).

### Neurogenesis in the Subventricular Zone and Olfactory Bulb

#### The Stages of Subventricular Zone Neurogenesis

The lateral ventricles are lined with an abundance of neural stem cells (NSCs) referred to as type-B1 cells, which resemble astrocytes and differentiate into neurons that populate the OB (Doetsch et al., 1997, 1999; García-Verdugo et al., 1998; Mirzadeh et al., 2008; Ponti et al., 2017). Type-B1 cells express GFAP, glutamate aspartate transporter, and brain lipid-binding protein (Doetsch et al., 1997; Codega et al., 2014; Mich et al., 2014). Activated type-B1 cells express nestin and divide asymmetrically for self-renewal or to give rise to achaete-scute homolog 1- and distal-less homeobox 2-expressing C cells (Doetsch et al., 1997; Ponti et al., 2017). Type C cells divide symmetrically two to three times, subsequently differentiating into type A cells (neuroblasts), which represent the final stage of differentiation within the SVZ (Ponti et al., 2017). Type A cells divide one to two times and migrate through the rostral migratory stream (RMS) towards the OB (Doetsch and Alvarez-Buylla, 1996; Lois et al., 1996; Wichterle et al., 1997; Ponti et al., 2017). These cells express the microtubule-associated protein DCX as well as collapsin-response mediator protein-4, which is involved in axonal guidance. These proteins together allow the newly generated neuroblasts to interact with microtubules and myosin II, allowing them to travel at a speed of 120 μm/h towards the OB (Wichterle et al., 1997; Francis et al., 1999; Nacher et al., 2000). PSA-NCAM, ganglioside 9-O-acetyl GD3, and a host of integrins are also expressed by type A cells and have been shown to be involved in the migratory process, while Tenascin C is one of the extracellular matrix molecules in the RMS that interact with the integrins and 9-O-acetyl CD3 present in these immature neurons (Tomasiewicz et al., 1993; Yokosaki et al., 1996; Jacques et al., 1998; Probstmeier and Pesheva, 1999; Chazal et al., 2000; Miyakoshi et al., 2001; Murase and Horwitz, 2002; Emsley and Hagg, 2003). Notably, the directionality of this migration is regulated by a series of factors, including SLIT-roundabout (ROBO) signaling. SLITs are chemorepulsive to type A cells, and ROBO receptors are expressed in the SVZ and the RMS (Ba-Charvet et al., 1999; Hu, 1999; Li et al., 1999; Wu et al., 1999). A gradient of SLITs is established by cilia, with the highest concentration being in the SVZ, driving type A cells away (Sawamoto et al., 2006). Following directional migration to the OB, neuroblasts proceed to migrate radially through a process regulated by factors such as tenascin-2 and prokineticin-2, which induce detachment from the RMS chains. Subsequently, type A cells integrate into the granule cell layer of the OB where they are thought to participate in plasticity and OB-dependent learning (Barnea and Nottebohm, 1994; Lois and Alvarez-buylla, 1994; Kempermann et al., 1997; Gould et al., 1999a). Only a very small portion of these cells survive to become mature granule cells (Lois and Alvarez-buylla, 1994). Those that survive tend to establish connections to mitral or tufted cells, which are relatively active (Petreanu and Alvarez-Buylla, 2002). It is also believed that BDNF acts as the main trophic factor required for survival and subsequent maturation into granule cells (Kirschenbaum and Goldman, 1995; Pencea et al., 2001a).

#### Regulation of SVZ Neurogenesis

Neurogenesis in the SVZ is regulated by a series of growth factors, signaling pathways, and neurotransmitters. The main growth factors involved in SVZ neurogenesis include the mitogens fibroblast growth factor 2 (FGF-2) and the epidermal growth factor 2, both of which are expressed by astrocytes and provide proliferative signals to the NSCs in the SVZ (Morita et al., 2005). Other growth factors involved include ciliary neurotrophic factor (CNTF, which is involved in NSC self-renewal), vascular endothelial growth factor (VEGF, important for angiogenesis), pigment epithelium-derived factor (involved in type-B1 cell maintenance), and betacellulin (which increases proliferation; Palmer et al., 2000; Jin et al., 2002; Emsley and Hagg, 2003; Greenberg and Jin, 2005; Ramírez-Castillejo et al., 2006; Gómez-Gaviro et al., 2012). Conversely, endothelial cells secrete neurothrophin-3 (NT-3), which leads to nitric oxide production, a cytostatic factor (Delgado et al., 2014).

Additionally, various neurotransmitters and neurotransmitter-related proteins play a role in the modulation of SVZ neurogenesis. For example, serotonin (5-HT) terminals are thought to form a dense plexus that modulates ependymal and type-B1 cells. As such, serotonergic neurotransmission may play a critical role in the initial stages of SVZ cell proliferation (Banasr et al., 2004; Tong et al., 2014). GABA can inhibit both cell proliferation and neuronal differentiation in this neurogenic region. However, type-B1 and type-C cells express the diazepam-binding inhibitor protein, which competitively inhibits the binding of GABA to its receptors, thus preventing GABAergic neurotransmission and promoting cell proliferation (Liu et al., 2005; Fernando et al., 2011; Alfonso et al., 2012). Lastly, the population of cholinergic neurons (which expresses choline acetyl transferase) present in the SVZ is also thought to regulate neuroblast proliferation through activation of fibroblast growth factor receptor (FGFR)-mediated signaling (Paez-Gonzalez et al., 2014). Recently, it has been suggested that the hormone ghrelin might also have a role in cell proliferation and neuroblast formation in the SVZ (Li et al., 2014).

#### Functions of SVZ Neurogenesis

The functional significance of SVZ neurogenesis has been less well characterized when compared to that of hippocampal neurogenesis. Nevertheless, SVZ neurogenesis occurs throughout adulthood in the mammalian brain and largely contributes to the development of optimal olfactory circuitry (Gheusi et al., 2000; Cecchi et al., 2001). Through constant granule cell regeneration and replacement, mammals are able to respond to new environmental stimuli and to reinforce particular odorant representations that are more pervasive in their environment (Alvarez-Buylla and García-Verdugo, 2002; Lim and Alvarez-buylla, 2016). There is further evidence that steroid hormones may also have an influence in SVZ/OB neurogenesis, suggesting a role in sexual function (Lau et al., 2011). In particular, steroid hormones (namely, estrogen) may be involved in the survival of newly generated OB neurons, allowing for the potential regulation of sexual behavior (Rasika et al., 1994; Burek et al., 1995; Hidalgo et al., 1995; Tanapat et al., 1999).

## “Novel” Adult Neurogenic Zones

### Neurogenesis in the Hypothalamus

#### Neurogenic Niches in the Hypothalamus

Evans et al. (2002) provided the first evidence of neurogenesis in the hypothalamus, particularly around the third ventricle. Adult rat neurons from the hypothalamus were cultured and, within 7 days, two cell lines developed (Evans et al., 2002). One group of cells was positive for GFAP, while the second expressed alpha-internexin. These alpha-internexin-positive cells underwent mitosis, expressed several neuronal markers, produced two-dimensional cellular networks, and had electrophysiological properties consistent with those of newly generated neurons (Evans et al., 2002). Two years later, using a similar model, Markakis et al. (2004) isolated three categories of newly generated cells from the hypothalamus, including a population of cells expressing dopamine, gonadotropin-releasing hormone, thyrotropin-releasing hormone, oxytocin, and vasopressin. This proved to be a landmark study, as it demonstrated the neurogenic potential of the hypothalamus (Markakis et al., 2004).

Subsequent studies have determined a number of neurogenic niches within the hypothalamus as well as regulatory factors and functions of hypothalamic neurogenesis. Using both male and female rats, Xu et al. (2005) demonstrated that the ependymal cells of the third ventricle retain a low but noticeable neurogenic potential between post-natal days (PNDs) 56 and 63. These ependymal cells were labeled by the exogenous proliferation marker 5-bromo-2′-deoxyuridine (BrdU), and their proliferation could be enhanced by FGF-2 (Xu et al., 2005). Furthermore, this study demonstrated that neurospheres obtained from this brain region could be grown *in vitro*. One subpopulation of neuroprogenitor cells was identified as being tanycytes, which line the third ventricle. By engineering these cells to express green fluorescence protein (GFP), the authors were able to show their migration and integration into neural networks in various regions within the hypothalamic parenchyma (Xu et al., 2005).

Pérez-Martín et al. (2010) further evaluated the wall of the third ventricle in adult male and female rats (PNDs 56–63) and proposed its subdivision into three subregions of varying degrees of proliferation: a non-proliferative dorsal zone, a middle third of subependymal cells, and a ventral zone containing tanycytes (Pérez-Martín et al., 2010). Tanycytes exhibited greater basal growth capacity than subependymal cells, but both had inferior proliferative capacity when compared with proliferating cells in the SVZ. Both hypothalamic subregions were responsive to insulin-like growth factor 1 (IGF-1), and thus it was concluded that these could be considered IGF-1-responsive neurogenic niches (Pérez-Martín et al., 2010). Interestingly, a subset of DCX-positive neuroblasts was shown to migrate to the ventromedial nucleus of the hypothalamus (VMH) where they express Hu, a mature neuronal marker (Batailler et al., 2014). Notably, the presence of DCX-positive neuroblasts was detected (in slightly different distributions) in the hypothalamus of mice, sheep, and humans (Batailler et al., 2014).

More recently, the median eminence (ME) has emerged as the hypothalamic region with the most potent neurogenic capacity. Lee et al. (2012) described the rate of neurogenesis in the mouse ME as being fivefold higher than that seen in other hypothalamic regions (Lee et al., 2012). This neurogenic niche is also comprised of tanycytes, and a long-term diet-responsive effect has been observed in this region (Lee et al., 2012). Thus, when comparing mice fed a HFD with those on normal chow, there was no change in energy balance at 35 or 45 days, but when HFD mice reached PND75 (i.e., adulthood), their neurogenesis rate quadrupled as indicated by the increased expression of BrdU/Hu-positive cells. This overfeeding-triggered neurogenesis appeared to be correlated with a reduction in metabolic rate and weight gain through increased fat storage.

Contrarily, McNay et al. (2012) examined neurogenesis in the energy-balancing circuit of the hypothalamic arcuate nucleus (ARN) in two mouse models of obesity: leptin deficiency and HFD-induced obesity (DIO). Interestingly, in DIO mice, an increase in the number of stem cells was observed within 48 h; however, many of these cells failed to survive at the 4-week time-point. Further analysis revealed that, despite an overall increase in the number of hypothalamus NSCs, there was a reduction in the number of highly proliferative progenitors. Thus, it can be inferred that hypothalamic neurogenesis may be an acute response to metabolic stress. Indeed an HFD led to increase in retention of proopiomelanocortin (POMC)- and neuropeptide Y (NPY)-labeled neurons, whereas subsequent calorie restriction resulted in the normalization of the endogenous neurogenic rate. In leptin-deficient mice, there was an even greater loss of neurogenic function due to a severe deficiency in hypothalamic NSCs (McNay et al., 2012). The mechanisms through which leptin influences the rate of hypothalamic neurogenesis have not been fully elucidated, although studies suggest that neuropeptide Y may be an important player in that mechanism (Pierce and Xu, 2010).

Tanycytes can be subdivided into four main subtypes based on their position, gene expression profile, innervation, function, and neurogenic potential (Rodríguez et al., 2005; Robins et al., 2013; Recabal et al., 2017). These subtypes have been referred to as α1, α2, β1, and β2. It is the α2-tanycytes that specifically display neurogenic characteristics (Rodríguez et al., 2005; Robins et al., 2013). These α2 cells line the infundibular recess of the third ventricle and have direct contact with cerebrospinal fluid (CSF). α2 and β1 tanycytes project to the ARN to modulate the neuronal circuits involved in metabolism, whereas β2 tanycytes project to the ME and form a barrier between the CSF and the ME (Rodríguez et al., 2005). All four subtypes of tanycytes exhibit further characteristics of short-term metabolic regulation (Cortés-Campos et al., 2011; Orellana et al., 2012; Balland et al., 2014; Collden et al., 2015). Their influence on long-term metabolic regulation and how neurogenesis might impact this regulation are areas of ongoing research. Table 1 summarizes the studies that have evaluated neurogenesis in the hypothalamic brain region.

**Table 1:** Summary of studies investigating neurogenesis in the hypothalamus.

Species	Age/Sex	Manipulation/ Treatment	Proliferation	Survival/Differentiation	Reference
Wistar albino rats	2 months; Female (30) and Male (14)	IGF-1 at 12 h or 18 days post BrdU injection Male vs. female	↑BrdU–12 h; IGF-1	**IGF-1 injection**: ↑: BrdU–18 days, GFAP, NeuN	Pérez-Martín et al. (2010)
Mice	8 weeks; Male	CNTF HFD Ara-C	-	**No CNTF**: ↑: BrdU–60–72 h, Hu **CNTF injection (compared to control)**: ↑: BrdU–60–72 h, Hu, TuJ1, DCX, NPY, POMC **Ara-C injection:** Blocked CNTF induced cell proliferation	Kokoeva (2005)
C57BL/6 mice	7 weeks; Male	HFD Ara-C	-	**Normal diet**: ↑: BrdU—Day 2–5, Ki67–1 day, GFAP, Iba-1 **HFD (compare to control)**: ↑: BrdU—Day 2–3, Ki67–Day 1 + 3, GFAP, IBa-1, POMC **↓**: BrdU—Day 4–5, Ki67 to control levels—Day 5 **Ara-C injection:** No production of BrdU	Gouazé et al. (2013)
Nestin-Cre-R26 mice	35 days and 75 days; Female	Age HFD	-	**HFD mice 75d**: ↑: BrdU, Hu, NPY, POMC	Lee et al. (2012)
C57BL/6 mice FVB mice Ob/ob mice NPY-hrGFP mice WT C57BL/6 mice	16 weeks; Male and Female	HFD vs. Calorie restrictions	-	**↑**: BrdU, Hu, NeuN, POMC **HFD (compared to control):** ↓: BrdU **=**: Proportion Hu **Calorie restriction (post HFD):** ↑BrdU 69% ↑NPY ↑POMC **Leptin deficient (ob/ob):** ↓BrdU =Hu	McNay et al. (2012)
Fgf10n^lacZ/+^ Fgf10^CreERT2^: R26^lacZ^ Fgf10^CreERT2^:R26^Tom^	-	Fed vs. fasted	-	**Amongst Fgf10-Tanycytes:** (−) NeuN (−) GFAP (−) S100B (+) Olig2 persists with age **↓**Tanycytes with age **Amongst X-gal cells:** (+)NeuN	Haan et al. (2013)
C57BL/6 mice	6 weeks; Female and Male	HFD vs. LPD (low protein diet) HFD vs. caloric restriction Female vs. male	-	**Female HFD and LPD (compared to normal chow)**: ↑: Hu in ME, BrdU—ME **↓**: Hu in ArcN, BrdU—ArcN **Female caloric restriction (compared to HFD):** ↓ BrdU **Male HFD (compared to female HFD and NC):** ↓ :ArCN neurogenesis, ME neurogenesis	Lee et al. (2014)
C57BL/6 mice	10–12 weeks; Female	Ovariectomized and implanted with E2 vs. oil HFD	**HFD:** ↑BrdU mArcN, pArcN, mVMH **E2:** ↓ BrdU	↑ERα ↑pSTAT3 **HFD (compared to control):** ↑BrdU ↑ERα ↑pSTAT3 **E2:** ↓ ERα	Bless et al. (2016)
Nestin^CreERT2^: CAG-R26^tdTomato/+^: IGF-1R^flox/flox^	12 weeks; Male	IGF-1R	-	↑α-tanycytes Fewer gliogenic **9 months:** ↑neurons PH, DMH, VMH **IGF-1R KO:** ↑NeuN	Chaker et al. (2016)
*Swiss albino mice*	5 weeks; Male	**HFD for 8 weeks then:** Protocol 1.- HFD vs. HFD w/partial substitute **Protocol 2** - HFD vs. HFD w/substitute vs. NFD vs. NFD w/supplementation **Protocol 3**–Saline vs. BDNF vs. DHA **Protocol 4**–Saline vs. DHA vs. high dose DHA	**↑** BrdU in BDNF and DHA groups	**HFD w/substitute:** ↑ DCX ↓Bax, Bax-BCL-2 ratio **HFD:** ↑Bax **DHA:** ↑BrdU/NeuN, POMC	Nascimento et al. (2016)
Fgf10n^lacZ/+^ Fgf10^CreERT2^: R26^lacZ^ Fgf10^CreERT2^:R26^Tom^	-	Fed vs. fasted	-	**Amongst Fgf10-Tanycytes:** (−) NeuN (−) GFAP (−) S100B (+) Olig2 persists with age **↓**Tanycytes with age **Amongst X-gal cells:** (+)NeuN	Haan et al. (2013)
C57BL/6 mice	6 weeks; Female and Male	HFD vs. LPD (low protein diet) HFD vs. caloric restriction Female vs. male	-	**Female HFD and LPD (compared to normal chow)**: ↑: Hu in ME, BrdU—ME **↓**: Hu in ArcN, BrdU—ArcN **Female caloric restriction (compared to HFD):** ↓ BrdU **Male HFD (compared to female HFD and NC):** ↓ :ArCN neurogenesis, ME neurogenesis	Lee et al. (2014)
C57BL/6 mice	10–12 weeks; Female	Ovariectomized and implanted with E2 vs. oil HFD	**HFD:** ↑BrdU mArcN, pArcN, mVMH **E2:** ↓ BrdU	↑ERα ↑pSTAT3 **HFD (compared to control):** ↑BrdU ↑ERα ↑pSTAT3 **E2:** ↓ ERα	Bless et al. (2016)
Nestin^CreERT2^: CAG-R26^tdTomato/+^: IGF-1R^flox/flox^	12 weeks; Male	IGF-1R	-	↑α-tanycytes Fewer gliogenic **9 months:** ↑neurons PH, DMH, VMH **IGF-1R KO:** ↑NeuN	Chaker et al. (2016)
*Swiss albino mice*	5 weeks; Male	**HFD for 8 weeks then:** Protocol 1.-HFD vs. HFD w/partial substitute **Protocol 2**-HFD vs. HFD w/substitute vs. NFD vs. NFD w/supplementation **Protocol 3**–Saline vs. BDNF vs. DHA **Protocol 4**–Saline vs. DHA vs. high dose DHA	**↑** BrdU in BDNF and DHA groups	**HFD w/substitute:** ↑ DCX ↓Bax, Bax-BCL-2 ratio **HFD:** ↑Bax **DHA:** ↑BrdU/NeuN, POMC	Nascimento et al. (2016)

#### Modulation of Hypothalamic Neurogenesis

As in other brain regions, various trophic factors have emerged as potent stimulators of hypothalamic neurogenesis. For example, BDNF was shown to increase the number of BrdU-labeled cells in the rat hypothalamus. Furthermore, among the BrdU-labeled neurons, a subset was shown to co-express the neuronal markers TuJ1 (class III β-tubulin) and microtubule-associated protein 2 (Pencea et al., 2001b). Another growth factor, IGF-1, has also been shown to stimulate hypothalamic neurogenesis, an effect that is not surprising, bearing its metabolic function. Thus, IGF-1 treatment has been associated with the production of new cells in the subependyma, and the new tanycytes were shown to co-express BrdU and NeuN (Pérez-Martín et al., 2010). Various studies have also assessed the effects of certain pharmacologic manipulations, namely, neuroleptics, on cell proliferation and neuroblast formation in the adult rat hypothalamus. These studies reported that the long-term, but not short-term, administration of olanzapine, chlorpromazine, and haloperidol can increase the number of Ki-67-positive cells in the hypothalamus of adult male rats (Rojczyk et al., 2015). With regards to neuronal differentiation (i.e., DCX expression), the results were not consistent and seemed to depend on the drug used and the regime of administration. Indeed single injections of chlorpromazine and olanzapine decreased the number of hypothalamic neuroblasts, whereas the long-term administration of chlorpromazine increased the neuroblast number, but the long-term administration of haloperidol decreased neuroblast formation (Rojczyk et al., 2015). Voluntary exercise has also been shown to increase the number of BrdU-positive cells that resembled tanycytes in the rat hypothalamus, and this was accompanied by an increase in the expression of FGF-2 and FGFR in the ependymal and the subependymal layers (Niwa et al., 2016). Finally, in response to electrolytic lesioning of the hypothalamus, an increase in new BrdU-labeled cells was observed in the SVZ. This increase persisted for at least 30 days post-injury, and some of the newly generated cells were shown to migrate toward the hypothalamus, indicating that these two neurogenic regions are likely interconnected (Cao et al., 2002).

#### Functional Roles of Hypothalamic Neurogenesis

*Via* its outputs to the pituitary gland, the hypothalamus serves several key neuroendocrine, sexual, and physiologic functions by modulating downstream targets such as the adrenal cortex, thyroid, bones, muscles, sexual organs, and kidneys (Tsigos and Chrousos, 2002). Therefore, many studies have investigated the role of hypothalamic neurogenesis on these functions. The following section will discuss the two most well-studied functional implications of hypothalamic neurogenesis: metabolic function and behavioral/sexual function (Lee and Blackshaw, 2012; Recabal et al., 2017).

#### The Role of Hypothalamic Neurogenesis in Metabolic Function

A number of recent studies have elucidated the role of hypothalamic neurogenesis in metabolic regulation. These effects are primarily exerted in the cells lining the third ventricle and mainly in the ARN, but also in the ME.

In an initial study, Kokoeva (2005) investigated the effects of CNTF (a protein known to induce weight loss in both obese rodents and humans) infusion in obese adult mice. Surprisingly, the authors noted a strong presence of newly generated neurons in the walls of the third ventricle (positive for both BrdU and CNTF), and these effects persisted for 2 weeks after CNTF infusion. Furthermore, this effect appeared to be mediated by an interaction between leptin and signal transducer and activator of transcription 3 (STAT3), the newly generated cells in the ARN expressed both POMC and NPY, and inhibition of mitosis through treatment with cytosine-beta-D-arabinofuranoside (AraC, an antimitotic agent) limited the long-term effects of CNTF on neurogenesis (Kokoeva, 2005). A subsequent study by Pierce and Xu (2010) largely confirmed the results of Kokoeva (2005) by testing the effects of acute vs. gradual agouti-related protein ablation (AgRP, an orexigenic factor). These authors found that the acute ablation of AgRP led to weight loss and decreased food intake due to decreased orexigenic drive but that gradual ablation had no metabolic or feeding effect—suggesting a compensatory mechanism through hypothalamic neurogenesis. A subset of neurons within the gradual ablation group became AgRP-positive and leptin responsive. The prevention of neurogenesis with AraC treatment led to a decrease in feeding and reduced body fat. Thus, AgRP is believed to play a role in energy homeostasis (Pierce and Xu, 2010). These results, combined with those previously described in the study of Lee et al. (2012), further support an indirect (STAT3-mediated) effect of leptin on hypothalamic neurogenesis (Lee et al., 2012). To further investigate the role of hypothalamic neurogenesis in food intake, Gouazé et al. (2013) evaluated the effects of central administration of the antimitotic agent AraC in adult mice under HFD for 3 weeks (Gouazé et al., 2013). HFD led to an increased number of BrdU-positive cells in the ARN that lasted up to 3 days, followed by a subsequent reduction in cell number. In addition, blocking cell proliferation with AraC led to increased weight gain on HFD, suggesting that hypothalamic neurogenesis protects against excessive weight gain (Gouazé et al., 2013).

In a subsequent study, Haan et al. (2013) evaluated whether fibroblast growth factor 10 (FGF10, known to play a role in maintaining progenitor cell function in neural and non-neural contexts) would be involved in early tanycytic neurogenic response to appetite and energy balance (Haan et al., 2013). Notably, these authors found that most cells in the ME and parenchyma of postnatal and adult mice do not have FGF10-expressing progenitors and that FGF10 alpha-negative tanycytes do not proliferate. On the other hand, FGF10-positive cells did show neurogenic function and became scarcer with age, thus highlighting the role of this factor in hypothalamic (tanycytic neurogenesis; Haan et al., 2013). Robins et al. (2013) further investigated the role of alpha subtype tanycytes in hypothalamic neurogenesis and the effect of variable gene expression on tanycyte cell function (Robins et al., 2013). In adult mice (P42–56), a number of subsets of α-tanycytes were identified with variable gene expression and neurogenic function. Notably, α2-tanycytes were GFAP-positive (a marker of NSPC). Alpha tanycytes were shown to give rise to more α2-tanycytes as well as astrocytic cells. On the other hand, no evidence of neurogenic function was found in beta tanycytes. Further findings confirmed the responsiveness of alpha-tanycytes to FGF-2, which led to enhanced α2-tanycyte proliferation. In addition, this study also demonstrated that neurosphere location was correlated with tanycyte position and that, in particular, α2-tanycytes were located in neurospherogenic areas. All subtypes of alpha neurospheres (including α1, ventral α2, and dorsal α2) exhibited neurospherogenic function, but only α2-tanycytes showed stem cell-like characteristics with robust self-renewal. α2-tanycytes were characterized as being infrequently dividing stem-like cells with limited, but apparent, renewal potential (Robins et al., 2013). These results together suggest that tanycyte neurogenesis in the ME leads to weight gain and changes in metabolic function (Lee et al., 2013). In another study, Chaker et al. (2016) assessed how hypothalamic neurogenesis changed over time and was impacted by IGF-1 in male mice. These authors found a significant increase in ependymal cell density between 4 and 16 weeks (adulthood). and this was almost exclusively the result of an increase in alpha-tanycytes. Notably, at 9 months of age, the predominant new cell type in the ARC and ME was tdTomato-positive neurons (indicating recent proliferation). Furthermore, the newly integrated neurons were STAT3-negative and did not co-localize with NPY; however, some new neurons produced growth-hormone-releasing hormone and most had receptors for GABA or glutamate, suggesting that these were most likely interneurons. Moreover, deletion of the IGF-1 gene increased short- and long-term neurogenesis, indicating that the suppression of IGF-1 signaling could reduce the decline in hypothalamic neurogenesis associated with age (Chaker et al., 2016).

Lee et al. (2014) assessed the differential effect of a HFD, a low-protein diet (LPD), and a calorie-restricted diet on various neurogenic areas of the hypothalamus in male and female rats (Lee et al., 2014). First, in young adult female rats, HFD and LPD decreased the BrdU-labeled neurons in the ARN but increased the levels of new neurons in the ME. Conversely, a reduction in ME neurogenesis in response to calorie restriction in the ME was also observed despite a main effect of diet on ARN neurogenesis. Results in the ME demonstrated that there was a main effect of diet on ME neurogenesis and that there was a sex-diet interaction, such that female rats on HFD showed higher levels of neurogenesis than normal-chow-fed female rats. This effect, however, did not exist in males. Sex itself did not affect ME neurogenesis, and this sex–diet effect was not present in rats fed normal chow. Additionally, females had different rates of neurogenesis in the ME vs. in the ARN, while there was no difference in males between these two areas. Thus, HFD-dependent modulation of neurogenesis is sexually dimorphic, but only in the ME region. Consistent with these findings, blocking ME neurogenesis only reduced the HFD-induced weight gain in young adult female rats, but not in their age-matched male counterparts (Lee et al., 2014).

Given these differential effects of diet on hypothalamic neurogenesis in male and female rats, Bless et al. (2016) studied the effects of estrogen in regulating this process. Female P70–84 mice were bilaterally ovariectomized and randomized into either standard diet or HFD plus estrogen or vehicle (four groups in total; Bless et al., 2016). Animals treated with estrogen showed lower food intake than animals treated with vehicle, and HFD–vehicle animals weighed 35% more than those from the other treatment groups. The results showed that estrogen decreased neurogenesis in the anterior, medial, and posterior regions of the ARN and the anterior, medial, and posterior VMH. Furthermore, although HFD increased neurogenesis, this was attenuated by estrogen. All of these regions contained cells expressing BrdU and estrogen receptor (ER) alpha. In addition, HFD tended to increase the number of ER-expressing cells in the medial ARC and the medial VMH, and estrogen attenuated this effect. Some of the newly generated neurons were leptin responsive as indicated by STAT2 labeling. The number of ER-positive cells was greater in the medial ARC and the medial VMH of mice fed HFD, while the number of leptin-sensitive neurons in the entire VMH was increased by HFD. This had no effect on leptin-sensitive cells in the ARC. Additionally, these authors also reported that ER and STAT3 co-expressing cells were most dense in the medial ARC and that estrogen 2 (E2) in HFD-fed mice affects FGF10 gene expression, suggesting a mechanism by which estrogen can downregulate hypothalamic neurogenesis (Bless et al., 2016). Therefore, it can be speculated that differences in estrogen levels between male and female rats may underlie the sex-specific effects of HFD on weight gain (Lee et al., 2014).

Given the apparent role of hypothalamic neurogenesis in fat storage and metabolic regulation, Nascimento et al. (2016) sought to study the dietary influence of *n*-3 polyunsaturated fatty acids (PUFA) on hypothalamic neurogenesis (Nascimento et al., 2016). In 16-week-old mice, PUFA substitution for saturated fatty acids had the metabolic effects of reducing body mass, increasing caloric intake, and improving leptin response. This was manifested at the level of the hypothalamus by a further increase in hypothalamic neurogenesis above the level seen with HFD; however, PUFAs were found to primarily increase the levels of POMC-expressing hypothalamic neurons and not NPY-containing neurons, which is consistent with the metabolic findings. Furthermore, POMC was co-expressed with BDNF and GPR40 (a receptor for free fatty acids), while blockage of GPR40 blocked the neurogenic effects of PUFAs and blockage of BDNF led to a global reduction in hypothalamic neurons (Nascimento et al., 2016).

#### The Role of Hypothalamic Neurogenesis in Sexual/Mating Behaviors and Social Functions

The neurogenic and hormonal bases for social changes have been studied to a great extent. For example, hypothalamic neurogenesis in birds may influence social function, including song learning and mating. Bernstein et al. (1993) showed a recovery of courtship ability in male ring doves exposed to female birds following a hypothalamic lesion (Bernstein et al., 1993). In addition, female nest cooing has been shown to increase luteinizing hormone (LH) release (Cheng et al., 1998); moreover, estrogen affects IGF-1 receptor expression in the hypothalamus, and this is believed to have a role in LH surges (Pons and Torres-Aleman, 1993; Todd et al., 2010). Furthermore, courtship interactions lead to GnRH production in males (Mantei et al., 2008), which may influence neurogenesis in the hypothalamus.

Cheng et al. (2004) examined the effects of acoustic stimuli in the hypothalamus of electrolytically lesioned ring doves. BrdU-labeled cells were present within the first month and matured into neurons at 2–3 months post-lesion (BrdU/NeuN-positive and GFAP-negative; Cheng et al., 2004). This was accompanied by mature patterns of electrical activity and restoration of coo-responsive units (Cheng et al., 2004). In fact, electrolytic lesioning in ring doves was shown to result in the increased production of BrdU/GnRH-positive neurons in the hypothalamus in both males and females. In addition, more new neurons were developed during the pre-laying reproductive phase, suggesting that they may play a role in the reproductive cycle (Cheng et al., 2011). Another study showed that female interaction propagated the neurogenic effects of electrolytic lesioning in the hypothalamus of adult male ring doves (Chen et al., 2006). Notably, this effect could be inhibited by blockage of cell mitosis with AraC treatment (Chen and Cheng, 2007). Fowler et al. (2002, 2005) obtained similar results in female prairie voles. Indeed male exposure increased the number of BrdU-labeled cells proliferating in the female prairie vole hypothalamus. Interestingly, BrdU-labeled hypothalamic cells also labeled positive for TuJ1 (Fowler et al., 2002). Notably, treatment with estrogen accentuated these changes in the hypothalamus, particularly in meadow voles (Fowler et al., 2005). Taken together, these studies indicate that indeed hypothalamic neurogenesis may play an important role in social interactions in general and mating behaviors in particular, at least in birds. Further studies are warranted to determine whether the same is true in mammals, including humans.

### Neurogenesis in the Striatum and the Substantia Nigra

#### Generation of New Neurons in the Striatum

The heterogeneity of striatal neurons has been described for many years. In 1995, Parent et al. described a small subpopulation of CR-expressing neurons, including large branching neurons and medium-sized round neurons, with limited connectivity (Parent et al., 1995). These CR-expressing neurons have become an area of interest and have been reported in varying amounts in the striatum of rats, monkeys, and humans. Notably, these striatal neurons can be generated postnatally, as it was demonstrated using carbon-14 (C^14^) labeling (Spalding et al., 2005).

Indeed the presence of postnatally generated CR-positive neurons was first demonstrated in 1999 (Schlösser et al., 1999). By using immunohistochemistry for CR and parvalbumin (PV), the authors were able to show that, after birth, there is an increase in this population of CR-expressing neurons in the lateral striatum of rats. This increase peaked at 5 days and then decreased over the next 2 weeks. Notably, the number of PV-positive cells also increased up to 2–3 weeks following birth (Schlösser et al., 1999). Since these initial observations, several studies have described the migration of neurons from the SVZ to the striatum in multiple animal models. Notably and in agreement with these animal findings, a human transcriptome analysis revealed DCX levels in the striatum that were comparable to those seen in the hippocampus (Kang et al., 2011). These results were further supported by western blot analysis, showing that DCX and PSA-NCAM protein levels were comparable between the striatum and the hippocampus (Tong et al., 2011).

Using the isotope C^14^, the ground-breaking study by Ernst et al. (2014) provided further insight into the occurrence of striatal neurogenesis in the human brain and how this varies from that seen in animal models. This retrospective birth dating study utilized human brain samples from the Cold War era, during which atmospheric C^14^ increased, so as to use C^14^ to establish time of DNA synthesis. This study identified five main cell subtypes in the striatum, including DCX-positive cells, which often co-localized with CR and NPY. Of these NPY- and CR-expressing cells, about 20% were devoid of or contained low lipofuscin, an indicator of early cell age. Of the five subtypes of neurons, the medium-sized spiny neurons demonstrated C^14^ levels consistent with presence at birth, thus suggesting no neurogenic potential. The four types of interneurons contained C^14^ correlating with time-points after birth, demonstrating post-natal neurogenesis. Furthermore, non-neuronal cells (e.g., oligodendrocytes) also exhibited C^14^ levels, suggesting gliogenesis after birth. Interestingly, this study also assessed neurogenic potential in specimens from individuals with HD. Retrospective dating of neurons from HD striatal samples showed lower turnover rates in general, and in particular, grades 2 and 3 HD patients presented C^14^ levels that indicated no generation of new neurons after birth (Ernst et al., 2014).

#### Modulation of Striatal Neurogenesis

A variety of animal models have also pointed to a number of regulatory factors that enhance or inhibit striatal neurogenesis. FGF-2 and endothelial growth factor (EGF) were originally shown to increase BrdU/NeuN double-labeling following ischemia (Yoshikawa et al., 2010). On the other hand, parallel studies have indicated that the increased expression of the pro-neurogenic trophic factors BDNF (Im et al., 2010) and nerve growth factor (Zhu et al., 2011) is associated with functional recovery following ischemia. Moreover, overexpression of Bcl-2 was also shown to reduce the negative effects of ischemia while enhancing striatal neurogenesis (Lei et al., 2012).

Notably, various drugs have also been shown to increase striatal neurogenesis. For example, methamphetamine was shown to induce a low level of neurogenesis in the striatum of rats (Tulloch et al., 2014). In addition, an increase in the number of BrdU-labeled neurons co-expressing various striatal neuronal markers (including ChAT, PV, and dopamine- and cAMP-regulated phosphoprotein of molecular weight 32 kDa, DARPP-32) was also observed in the striatum of methamphetamine-treated mice. This methamphetamine-induced increase in striatal neurogenesis may reflect an endogenous compensatory mechanism to partially counteract striatal neuronal death induced by this drug (Tulloch et al., 2014). Lastly, the antidepressant pramipexole was also shown to increase the number of DCX-labeled neuroblasts in the dorsal region of the striatum (Salvi et al., 2016).

#### Ischemia/Stroke and Striatal Neurogenesis

A number of studies have described the heterogeneous nature of SVZ stem cells as well as their potential in contributing to the generation of new striatal neurons (Suzuki and Goldman, 2003; Young et al., 2007; Shapiro et al., 2009). Indeed through retrovirus-mediated GFP expression, new SVZ cells have been shown to migrate in several directions, including towards the striatum, resulting in the subsequent production of interneurons and non-neuronal cells (Suzuki and Goldman, 2003). In agreement, Inta et al. (2008) also showed SVZ cell migration to the striatum, cortex, and amygdala (Inta et al., 2008). BrdU and DCX labeling confirmed the neuronal fate of these cells, which were shown to develop into GABAergic interneurons. New striatal neurons were also shown to express various neuronal markers, including DCX, CRMP4, and NeuN, as well as neuron-specific enolase, glutamic acid decarboxylase (GAD-67), and CR (Dayer et al., 2005). Dayer et al., 2005 investigated the neuronal fate of SVZ BrdU- and DCX-positive cells in mice and showed a combination of migratory and newly integrated striatal cells. After 4–5 weeks, these BrdU-labeled cells expressed NeuN, Gad-67, and CR, resided mostly in the nucleus accumbens and the dorsomedial striatum, and were GABAergic (Dayer et al., 2005). Notably, empty spiracles homeobox 1 (Emx-1) transgenic mice revealed a further contribution of the Emx-1 lineage to the development of medium-sized spiny neurons in the striatum; however, this did not persist into adulthood (Gorski et al., 2002; Cocas et al., 2009). Similar findings have also been observed in squirrel monkeys. BrdU labeling in 4–6-year-old monkeys showed a large number of newly generated neurons in the striatum, with 5–10% co-expressing NeuN and suggesting that neurogenesis in this brain structure persists throughout adulthood (Bédard et al., 2002a). Furthermore, these neurons expressed factors involved in neuronal commitment and maturation. Notably, BrdU labeling revealed that these neurons were likely originated in the SVZ but deviated from the RMS, thus never reaching the OB and instead completing their differentiation and maturation in the striatum (Bédard et al., 2002b).

Interestingly, ischemic/stroke animal models generally show increased neurogenic potential in the striatum. This has been suggested as a method of self-repair following an ischemic event affecting the striatum. In a ground-breaking study, Arvidsson et al. (2002) used double-labeling with BrdU and DCX or NeuN and found a marked increase in proliferation and striatal recruitment of SVZ neuroblasts following occlusion of the middle cerebral artery in rats. Indeed a large number of these cells migrated into the striatum, some of which developed into striatal medium-sized spiny neurons or other mature neurons as indicated by the expression of Meis homeobox 2, Pbx homeobox, and DARPP-32. This study also demonstrated that AraC treatment markedly reduced the number of BrdU/DCX-positive cells in the striatum (Arvidsson et al., 2002). In a different study, GFAP labeling of SVZ cells after focal cerebral ischemia also demonstrated that the SVZ is indeed a source of striatal neuroblasts following an ischemic injury. After injury, a transient increase in DCX-positive cells and in long migratory neuroblast chains associated with striatal blood vessels was noted. Notably, these migrating neuroblasts proceeded to mature, express presynaptic vesicles, and form synapses within the striatal neurocircuitry (Yamashita et al., 2006). However, new migrating SVZ neurons have been shown to associate with both newly formed as well as old blood vessels in the post-stroke striatum, which means that these blood vessel-associated migratory pathways may not be essential (Kojima et al., 2010). Nevertheless, these newly produced GABAergic and cholinergic striatal neurons have been shown to develop dendrites, spines, and electrophysiological properties indicative of full integration into the pre-existing neuronal network of the striatum (Hou et al., 2008). That being said, some studies have also found that newly developed neurons may not fully replace the full spectrum of neurons lost due to ischemic injury. For example, a study using a neonatal rat model of hypoxia/ischemia by Yang et al. (2008) suggested that all newly generated neurons in the striatum are CR-positive but not positive for DARPP-32, calbindin, PV, somatostatin, or choline acetyltransferase (Yang et al., 2008). A later study also noted that NSCs from the SVZ give rise to striatal interneurons that express CR and Sp8 (a marker for mature striatal neurons) that persist long after DCX expression ceases. None of these newly generated neurons, however, expressed medium spiny neuron markers (Wei et al., 2011). The discrepancies among studies may reflect differences in the rodent models of stroke/ischemic insult used, the extent of ischemic striatal damage, as well as the age of the animals at the time of experimentation (which can impact their intrinsic neurogenic potential). Further studies are thus warranted in order to fully reconcile these results and determine the extent to which SVZ/striatal neurogenesis can be used as an endogenous strategy to replace the neurons lost following ischemic insults in rodents.

On the other hand, monkey models of ischemia showed lower neurogenic potential in response to stroke. While a significant increase in the number of newly generated neurons was observed following BrdU labeling in limited regions with variable presence in the striatum (Tonchev et al., 2003, 2005), a further analysis of the origin and the migration patterns of these neurons showed that these tended to migrate towards the OB, not the striatum. The striatum did, however, retain a small number of BrdU-positive cells (Tonchev et al., 2005).

#### Neurodegenerative Disorders and Striatal Neurogenesis

Nigrostriatal projections are implicated in neurodegenerative movement disorders, particularly HD and PD.

Table 2 summarizes the studies that have evaluated adult neurogenesis in the striatum and/or SN of HD and PD models. Notably, a number of studies have shown variable results with respect to the levels of endogenous neurogenesis in the HD striatum and how these can be modulated. In a quinolinic acid lesion model of HD in rats, an increase in SVZ proliferation was detected (as assessed with BrdU labeling), with newly developed neuroblasts migrating from the SVZ to the lesioned striatum (Tattersfield et al., 2004). However, in the R6/2 transgenic HD mouse model, no increase in the number of proliferating nuclear antigen (PCNA)-labeled cells was observed in the SVZ, and none of the detected BrdU-labeled cells co-expressed DCX or NeuN, indicating that, in this transgenic model, the striatum does not provide the necessary environment for the development of progenitor cells into mature neurons (Kohl et al., 2010). However, these transgenic HD mice did show an increase in the number of proliferating BrdU-labeled cells in the SVZ and the striatum in response to FGF-2 treatment. Notably, the new striatal cells recruited after administration of FGF-2 presented phenotypical features of medium-sized spiny neurons (as indicated by DARPP-32 immunolabeling), and this increase in endogenous striatal neurogenesis was accompanied by an improvement of functional outcomes in this animal model of HD (Jin et al., 2005). In another study, BDNF, in combination with Noggin (which suppresses gliogenesis), increased the number of BrdU/B-tubulin-III-positive neurons (which became DARPP-32 GABAergic neurons) and delayed disease progression in R6/2 transgenic mice (Cho et al., 2007). Moreover, when these mice were co-treated with AraC, these effects were negated due to the impaired production of new cells (Cho et al., 2007). Furthermore, in an excitotoxic model of HD, the development of striatal clusters of DCX- and/or Ki-67-positive cells that were closely associated with astrocytes was observed (Nato et al., 2015). Notably, a post-mortem analysis of the HD striatal subependymal layer also showed an increase in the expression of PCNA, β-tubulin-III GFAP, and NeuN-positive cells, suggesting that an increase in striatal HD neurogenesis may indeed be an endogenous compensatory mechanism in the HD striatum (Curtis et al., 2003).

**Table 2:** Summary of studies that have evaluated adult neurogenesis in the striatum and/or substantia nigra in models of Parkinson’s disease (PD; blue) or Huntington’s disease (HD; orange).

Species	Age/Sex	Manipulation/Treatment	Proliferation	Survival/Differentiation	Reference
C57BL/6 mice	3 mo;Female	Weight and age matched PPX (3.0 mg/Kg) vs ROP (3.0 mg/Kg)		**PPX:** ↑DCX;striatum	Salvi et al. (2016)
Sprague-Dawley Rats	Adult; Female	BDNF and Platelet-derived growth factor (PDGF) SN Lesion with 6-OHDA	**↑**BrdU	**3wk after Growth Factor treatment:** **↑**BrdU/NeuN **↓**BrdU/DARP-32	Mohapel et al. (2005)
Sprague-Dawley Rats	Adult; Female	6-OHDA lesions Intrastriatal LGF infusions	N/S	**↑**TH innervation in LGF infused group	Reimers et al. (2006)
C57BL/6 mice (from Harlan Sprague-Dawley)	8 wk	MPTP lesion	**↑**BrdU; SN and dorsal striatum	**↑**BrdU/GFAP	Kay and Blum (2000)
Sprague-Dawley rats	8-9 wk; Female	6-OHDA lesions FGF-2 FGF-8b	**↑**BrdU; SN	**↑**BrdU/Nestin **↓** BrdU w/ either B-tubulin III, NeuN, or TH; SN **FGF-2, FGF-8b:** **↑**B-tubullin III	Lie et al. (2002)
C57B1/6 mice	2-20 mo; Male	MPTP Lesion Ara-C	**↑**BrdU	**↑**BrdU/TH; SN 3 wk post lesion **Ara-C group:** **↓**TH cells	Zhao et al. (2003)
Sprague-Dawley Rats	250 g; Female	Injections of 7-OH DPAT vs 0.9 saline	N/S	**↑**BrdU, BrdU/NeuN, BrdU/TH	Van Kampen and Robertson (2005)
Unspecified mouse species	60-80 days; N/S	pNes/Lacz transgenic mice MPTP Lesion	N/S	**↑** NeuN/LacZ ; Substantia Nigra **↑** BrdU	Shan et al. (2006)
C57BL/6J Mice	8-10 wk; Male	MPTP Lesion Exercise	N/S	**Exercise (compared to no exercise in both vehicle and MPTP groups):** **↑**TH-labelled Neurons	Smith et al. (2011)
C57B16 mice	8-12 wk; Female	MPTP lesion Levodopa treatment	**↑**BrdU	**↑**NG2 **↓**TH cells	Klaissle et al. (2012)
Mice	3 mo; N/S	Th^lox^ mice, *nestin-CRE^ERT2^* mice*, SOX2-CRE^ERT2^* mice	N/S	**Th^lox^ mice, *nestin-CRE^ERT2^* mice:** **↓**TH cells	Albright et al. (2016)
C57bL/6 mice	25-40 g; N/S	Rgs5^GFP/+^ transgenic alteration Partial 6-OHDA PDGF-BB mini-osmostic pump	N/S	**6-OHDA group:** **↑**PDGFRβ+ **PDGF-BB group:** **↑** RGS5, NG2; VMS and DLS	Padel et al. (2016)
C57bL/6 mice Spraque-Dawley rats	9-10 wk; N/S 9-10 wk; N/S	6-OHDA lesion BDNF	N/S	**↓** BrdU/TH	Frielingsdorf et al. (2004)
C57BL/6 mice Sprague-Dawley rats Human Macaque monkey	10 wk; Female 10 wk; Male	Retroviral Labeling (GFP) MPTP lesion **Human:** PD and other neurological conditions	N/S	**↓** GFP/GFAP **↑**PSA, TH/PSA; SN	Yoshimi et al. (2005)
C57/B16 mice	40-60 d; N/S	6-OHDA Lesion Minocycline-mediated inhibition of neuroinflammation	N/S	**↑**DCX, GFAP; SN	Worlitzer et al. (2013)
Wistar Rats	Adult; Male	Quinolinic Acid (QA) lesion	**↑**BrdU; ipsilateral SVZ and lesioned striatum	**↑**BrdU and DCX; QA induced striatal lesion area, 20-30% BrdU/DCX	Tattersfield et al. (2004)
R6/2 Mice	Adult; Female	N/A	No difference from Wild Type Mice	**↑**DCX; SVZ neuroblasts in striatum	Kohl et al. (2010)
R6/2 Mice	N/S; Male and Female	Heterozygous Htt Exon-1 Transgenic FGF-2 stimulation	**FGF-2:** **↑**BrdU; SVZ,	**FGF-2:** **↑**DCX; SVZ	Jin et al. (2005)
R6/2 Mice	6wk, 4wk; N/S	BDNF/Noggin injections Ara-C	N/S	**BDNF/Noggin:** **↑**DARPP-32/BrdU, GAD67/BrdU. BrdU/BIII-tubulin **BDNF:** **↓** DARPP-32/BrdU, GAD67/BrdU	Cho et al. (2007)

***Acronyms and Abbreviations:** 6-OHDA, 6-hydroxydopamine, 7-OH DPAT, 7-hydroxy-diproprlaminotetralin; BAC, Bacterial Artificial Chromosome; BDNF, brain-derived neurotrophic factor; BrdU, Bromodeoxyuridine; DA, Dopaminergic; DCX, Doublecortin; FGF-2, Fibroblast Growth Factor 2; FGF-8b, Fibroblast Growth Factor 8b; GFAP, Glial Fibrillary Acidic Protein; GFP, Green Fluorescent Protein; HD, Huntingdon’s Disease; LGF, liver growth factor; MPTP, 1-Methyl-4-Phyenyl-1,2,3,6-Tetrahydropyridine; NeuN, Neuronal Nuclei; NG2, Neuro-Glial Antigen 2; PD, Parkinsons’s Disease; PDGF-BB, Platelet-Derived Growth Factor Receptor BB; PDGFβ+, Platelet-Derived Growth Factor Receptor-beta; PSA, Polysialic Acid; RGS5, Regulator of G-protein Signaling 5; SN, Substantia Nigra; TH, Tyrosine Hydroxylase; VMS, Ventro-Medial Striatum; DLS, Dorso-Lateral Striatum; wk, weeks; mo, months*.

With regards to PD, Kay and Blum (2000) were the first to demonstrate the existence of a population of BrdU-positive cells in the mouse striatum, which increased in number in response to 1-methyl-4-phenyl-1,2,3,6-tetrahydropyridine (MPTP) lesioning, an experimental model of PD. However, these cells only differentiated into GFAP-positive astrocytes (Kay and Blum, 2000). A 6-hydroxydopamine (6-OHDA) model of PD showed an increase in the number of BrdU/NeuN-labeled cells in the striatum, but none of these co-labeled for DARPP-32 (a marker of striatal neurons; Mohapel et al., 2005). Liver growth factor (LGF) likewise failed to stimulate neurogenesis in the striatum or SN of 6-OHDA-lesioned rats but did stimulate the outgrowth of neuronal terminals (Reimers et al., 2006). On the other hand, Porritt et al. (2000) found a new population of dopaminergic neurons expressing dopamine transporter (DAT) and tyrosine hydroxylase (TH) in post-mortem striatal samples of 10 PD patients, suggesting the existence of a potential endogenous compensatory mechanism in the PD striatum (Porritt et al., 2000; Table 2).

#### PD and SN Neurogenesis

The SN may contain NPCs, and if neurogenesis does occur in the SN, this may be decreased in the PD brain (Höglinger et al., 2004; Freundlieb et al., 2006; L’Episcopo et al., 2012). Kay and Blum (2000) first demonstrated the existence of a population of BrdU-labeled neurons in the SN of MPTP-lesioned male mice. However, within the SN, these cells remained undifferentiated (Kay and Blum, 2000). Lie et al. (2002) also observed a population of BrdU-positive cells in the SN; however, these progenitor cells preferentially differentiated into glial cells rather than giving rise to new neurons in this brain region (Lie et al., 2002).

Despite these initial observations, Zhao et al. (2003) found that the BrdU-labeled cells in normal mouse SN did eventually express the neuronal markers Hu and NeuN and developed synaptic connections to the striatum (Cassidy et al., 2003). Furthermore, it was suggested that dopamine itself (through the activation of dopamine D3 receptors) could induce the generation of new neurons (positive for BrdU, NeuN, and the dopaminergic marker TH) in the SN (Van Kampen and Robertson, 2005).

These findings, however, were not fully replicated in subsequent studies. Indeed using the exact same methods of the study of Zhao et al. (2003), Frielingsdorf et al. (2004) found no evidence of dopaminergic neuronal turnover in the SN of normal, hemiparkinsonian, and BDNF-treated rats (Frielingsdorf et al., 2004). In another study, newly proliferating cells in the SN of mice or rats (with or without MPTP treatment) were shown to actually express glial markers (Yoshimi et al., 2005). In agreement, Worlitzer et al. (2013) found that, while there was a population of DCX-expressing cells in the SN of 6-OHDA PD mice, these did not stain positive for TuJ1 or NeuN but instead resulted in gliogenesis. Notably, this effect was not affected by treatment with minocycline (Worlitzer et al., 2013). On the other hand, although an increase in BrdU and NeuN co-labeling was seen in the SN of a 6-OHDA PD rat model, none of the newly generated neurons developed into DARPP-32-positive dopaminergic neurons, arguing against the complete differentiation/maturation and functional integration of these newly generated neurons into the nigrostriatal circuitry (Mohapel et al., 2005). Moreover, although NPCs (that became positive for NeuN) were detected in the SN of a pNES-LacZ mouse model, the same was not found in normal mice (Shan et al., 2006). Furthermore, although MPTP lesioning was shown to increase the numbers of NPCs as well as TH- and NeuN-positive cells, these new SN cells were likely derived and migrated from other regions of the brain (Shan et al., 2006). Indeed it is possible that the new cells found in the SN actually developed from the ventricular system and that lesioning of dopaminergic neurons in the SN could increase the rate of SVZ (and not SN) precursor proliferation and neurogenesis (Cassidy et al., 2003).

The potential effects of various non-pharmacological and pharmacological therapeutic strategies on striatal (i.e., SVZ) and SN neurogenesis have also been assessed in different PD models. For example, exposure to treadmill exercise was found to further accentuate striatal/SN neurogenesis in both normal and MPTP-lesioned mice. Indeed exercise was shown to increase the number of TH-labeled neurons in the SN of MPTP-lesioned mice, an effect that was accompanied by functional recovery (Smith et al., 2011). In a different study, levodopa was also shown to increase BrdU- and nestin-labeled cells in the SN of MPTP mice, but these changes did not persist beyond 10 days. On the other hand, while exercise was not shown to have an effect on SN neurogenesis in this study, when exercise was used in combination with levodopa treatment, an increase in the number of BrdU/neural/glial antigen 2 (NG2)-positive cells was observed in the SN. Therefore, it appears that the effects of exercise on SN neurogenesis may be dopamine dependent (Klaissle et al., 2012). In addition, guanosine treatment was shown to decrease apoptosis, increase the number of dopaminergic neurons, enhance cell proliferation in the SN pars compacta, and improve motor function in a rat model of proteasome-inhibitor-induced parkinsonism (Su et al., 2009). Various endogenous factors have also been implicated in the modulation of SN neurogenesis. For example, Albright et al. (2016) found that a population of nestin-positive cells contributes to dopaminergic neuron turnover in this brain structure (Albright et al., 2016). Furthermore, using a 6-OHDA mouse model, Padel et al. (2016) also showed that platelet-derived growth factor (PDGF) can lead to the restoration of nigrostriatal fiber tracts (Padel et al., 2016).

### Neurogenesis in the Cortex

#### Identification of Neural Precursors in the Cortex

Although the cortex has not been classically considered as a “true” neurogenic area, various studies have indicated that neural precursor cells can be found in cortical areas. For example, Magavi et al. (2000) reported the presence of BrdU/NeuN-, BrdU/DCX-, and BrdU/Hu-positive neurons in the cerebral cortex after the induction of synchronous targeted apoptosis in layer VI of the cortex and that these new cells persist for at least 28 weeks post-injury (Magavi et al., 2000). In another study, neuroblast migration (i.e., presence of BrdU/DCX-positive cells) and maturation (i.e., presence of BrdU/NeuN-positive cells) were seen in damaged areas of the motor cortex (particularly in layer V). Notably, some of these newly generated motor neurons showed long-term survival (>56 weeks) and developed projections into the spinal cord (Chen et al., 2004). In another study, new neurons present in the motor cortex (as indicated by the co-expression of NeuN and NG2 in this brain region) were suggested to arise from both migratory neurons (originated in the SVZ) as well as precursor cells *in situ*. However, in this study, the new neurons appeared to be GABAergic interneurons rather than motor neurons (Dayer et al., 2005). The existence of a population of cortical progenitor cells was further confirmed by Tamura et al. (2007), who found NG2-positive cells co-expressing DCX in the rat neocortex. These authors further suggested that 1% of the newborn cells present in the neocortex committed to the neuronal lineage (TUC-4-positive), while approximately 10% of the progenitors differentiated into glial cells (Tamura et al., 2007).

Mixed results have been reported with regards to the neurogenic potential of the primate cortex. In macaque monkeys, BrdU labeling showed a limited presence of new neurons as well as glial cells (positive for GFAP) in the principal sulcus of the neocortex up to 12 weeks after BrdU treatment. Although the number of newborn cells did begin to decrease 9 weeks after BrdU injection, a small but significant number of NeuN-expressing cells was found in the neocortex at 2 and 9 weeks (Gould et al., 2001). However, a parallel study failed to replicate these findings and found no new neuronal production (as assessed with BrdU, GFAP, NeuN, and TuJ1 immunohistochemistry) in the principal sulcus of the neocortex 10–23 days after BrdU injection (Kornack and Rakic, 2001). Bernier et al. (2002) examined the piriform and the inferior temporal cortex of New World and Old World monkeys. NeuN, MAP-2, and TuJ1 labeling confirms that the BrdU-labeled cells in these regions expressed neuronal markers. These cells persisted to some extent by day 28 and were positive for Bcl-2 (which participates in the maturation of neuroblasts). However, evidence suggested that these newly generated cells were not native to the cortex but rather migrated from the SVZ (Bernier et al., 2002).

With regards to human studies, Arsenijevic et al. (2001) isolated progenitor cells from the adult human frontal and temporal cortex. In addition, the *in vitro* treatment of these progenitor cells with FGF-2 and EGF led to the formation of multipotent neurospheres, suggesting that indeed the adult human cortex possesses some neurogenic potential (Arsenijevic et al., 2001). However, a study using accelerator mass spectrometry to measure C^14^ levels in post-mortem tissue from individuals exposed to radioactivity found that none of the NeuN-expressing cells of the human cortex displayed the integration of this isotope. This finding suggests that new neurons in the human cortex may not fully differentiate during adulthood and that *in vitro* and *in vivo* data may not necessarily correlate (Bhardwaj et al., 2006). Furthermore, the occurrence of cortical stroke did not appear to induce or increase neurogenesis in humans in a similar analysis conducted several years later. Given these findings, the generation of new neurons in the human cortex is unlikely to continue into adulthood.

#### SVZ-Generated Neural Progenitors and Cortical Neurogenesis

Although some earlier studies in rodents were only able to identify the production of cortical glial cells from SVZ progenitors (Gould et al., 1999b; Suzuki and Goldman, 2003; Dayer et al., 2005), others reported evidence of SVZ-mediated cortical neurogenesis. For example, an increase in the expression of BrdU/MAP-2-labeled new neurons was found in the cortical layers of 12-week-old rats subjected to cortical stroke. However, these new neurons represented only a small portion of the new cells seen in the injured area (Gu et al., 2000). In another study, Jiang et al. (2001) found BrdU-positive cells co-expressing the mature neuronal markers MAP-2, β-tubulin III, or NeuN in the ischemic mouse cortex (Jiang et al., 2001).

Notably, at least part of these newly generated cells may have originated in the SVZ. Indeed, Inta et al. (2008) showed that SVZ BrdU-positive cells were able to populate the cortex and develop into GABAergic neurons. These newly generated neurons persisted up to 30 days after birth (Inta et al., 2008), with additional evidence suggesting that they may survive as long as 1 year (Osman et al., 2011). In addition, Jin et al. (2003) found that 90 min of focal ischemia resulted in cell migration from the RMS and the anterior SVZ into the cortex. A subset of these newly generated cells expressed nestin, suggesting neuronal differentiation; however, not all of these newly generated and migrating cells were neuronal (Jin et al., 2003). Cortical neurogenesis, maturation, and survival were also shown in serotonin (5HT) receptor 3a-enhanced green fluorescent protein (5HT3a-EGFP) transgenic mice in response to ischemic lesion. EGFP and BrdU co-labeling indicated an increase in SVZ neuroblast production at 2 weeks post-stroke, and these newly generated neurons were shown to populate the injured cortical region. Notably, labeling with caspase-3 (an apoptotic marker) indicated that only 3–5% of these cells underwent apoptosis by 35 days, while 50% of them expressed NeuN, indicating neuronal differentiation and maturation (Kreuzberg et al., 2010). Saha et al. (2013) further characterized the SVZ and cortical neurogenic responses to stroke. According to these authors, cortical lesion increased the number of SVZ cells expressing BrdU both ipsilaterally and contralaterally to the lesion, and this increase in proliferation peaked at 7 days. Notably, most of these cells deviated from the RMS and showed ectopic migration to the cortex *via* the corpus callosum. Most of this migration was guided along blood vessels or glial cells (Saha et al., 2013). A later study hypothesized that neurogenic changes in response to middle cerebral artery occlusion (MCAO) may vary longitudinally. Indeed a decrease in SVZ neurogenesis was observed 1 day post-infarct, whereas a peak increase in cell proliferation was noted at day 14, followed by a subsequent reduction at 28 days. In addition, this study also defined three potential destinations for the newly generated cells: Z1, the SVZ itself; Z2, along the corpus callosum; and Z3, the infarcted area of the cortex, with MCAO increasing migration to the Z2 and Z3 regions. The number of new neurons (BrdU/NeuN-positive) was high in the infarcted area between days 7 and 14, decreased by day 28, and then increased again 65 day post-stroke, suggesting a long-term sustained effect. Curiously, there was no difference between ischemic and control mice in terms of NeuN-expressing neurons, indicating that the newly generated neurons replaced the ones lost due to stroke (Palma-Tortosa et al., 2017). Mechanistically, this SVZ-mediated cortical neurogenesis is thought to be modulated by toll-like receptor 4 (TLR4). Indeed TLR4 was shown to play a key role in the migration of neurons from the SVZ to the cortex and their subsequent maturation, with TLR4-deficient mice presenting with increased levels of neuroblasts along the migratory path, without these ever reaching their final destination and maturation stage (Moraga et al., 2014). Table 3 provides a summary of the studies that have evaluated neurogenesis in the cortical region in models of cortical stroke/ischemia.

**Table 3:** Summary of studies investigating neurogenesis in the cortex.

Species	Age/Sex	Manipulation/ Treatment	Proliferation	Survival/Differentiation	Reference
C57BL/6 mice	Female	Photothrombotic stroke lesion in left hemisphere	N/S	**↑DCX** in striatum up to 6 weeks, and in peri-infarct area up to 1 year	Osman et al. (2011)
Wistar rats	12 weeks; Male	Reversible photothrombotic stroke lesion	N/S	BrdU/NeuN doubles in cortical region-at-risk at min 72 h.	Gu et al. (2000)
Wistar rats	9–10 weeks; Male	Middle Cerebral artery occlusion (MCAO) vs. SHAM vs. Control (no BrdU or MCAO)	N/S	**↑**BrdU/1 of MAP-2, B-tubullin III, and NeuN; 30–60 days after ischemic onset; Cortical layers II-VI	Jiang et al. (2001)
C57BL/10CsNJ and C57BL/10J mice	203 months; Male	Permanent MCAO Toll-like receptor 4 (TLR4) deficiency vs. wild-type	No change in TLR4 deficient group	**TLR4 deficient**: ↑Neuroblasts; all migratory zones **TLR2 ^+^/^+^**: ↑ NeuN/BrdU/GAD67 (interneuron markers); 14–28 days after stroke; peri-infarct cortex	Moraga et al. (2014)
Sprague–Dawley rats	280–310 g; Male	Right MCAO	N/S	BrdU/DCX in ischemic area DCX/NeuN in cortex	Jin et al. (2003)
5HT3A—EGFP transgenic mice	16–18 weeks; Male	MCAO	EGFP/BrdU	NeuN in 50% of BrdU/EGFP labeled SVZ neuroblasts—injured cortical region.	Kreuzberg et al. (2010)
C57BL/6 mice	4–6 months; Female and Male	Lesioning through aspiration in left motor cortex.	BrdU–ipsi/contralaterally in SVZ cells in cortex	BrdU/DCX in SVZ progenitor cells—corpus callosum and cortex adjacent to lesioned area	Saha et al. (2013)
C57BL/6 mice	2–3 months; Male	Focal ischemia	N/S	**↑** BrdU/NeuN—ischemic area days 7–14, and day 65	Palma-Tortosa et al. (2017)
Mice	6 weeks; Male	Cerebral ischemia	**↑**BrDu	Nestin—cortex ipsilateral to ischemia **7 days**: Nestin/s100-B, Nestin/NG2 **2 days-14 days**: Pax6	Nakagomi et al. (2009)
Human	34 years–84 years; both	Cardiogenic cerebral embolism	N/S	Nestin, Musashi-1–positive labeling at site of ischemic lesion β-tubulin III and GFAP– negative labeling at site of ischemic lesion	Nakayama et al. (2010)
Wistar rats	6 months; Male	Ischemic Insult	**Pre ischemic insult** ↑BrdU, PH3, Ki67/GFP/GAD67 Cortex Layer 1	**Post ischemic insult** GFL/GAD67 in Cortex layers 2–6	Ohira et al. (2010)
Long-Evans rats	90–110 days; Male	Focal permanent devascularisation, intraventricular infusions of EGF for 7 days, followed by EPO for 7 days in contralateral ventricle Control received aCSF infusions	N/S	**↑**NeuN from day 11–42 post stroke—Lesioned area	Kolb et al. (2007)
VEGF-Tg mice on C57BL/6 background	21–26 g; N/S	Vascular endothelial growth factor (VEGF) overexpressing mice vs. control MCAO	N/S	**↑** BrdU/Dcx in ipsilateral SVZ 14 days after MCAO Neuroblasts extending from SVZ to peri-infact cortex **↑**BrdU/NeuN 21 and 28 after MCAO	Wang et al. (2007)
Rats	Adult; Male	Induced MCAO Adeno-associated viral (AAV) vector delivery of Fibroblast growth factor 2 (FGF2) ARA-C injections	N/S	**↑** BrdU in 85% of lesioned cortex at 7 days post ARA-C **↑** BrdU/NeuN, BrdU/Hu in ischemia cerebral cortex **↑** SOX-2 at 30 days post-ischemia – ventricular region **AAV-FGF group** ↑ BrdU/NeuN, BrdU/Hu compared to non-FGF group 2× **↑** SOX-2 at 30 days post ischemia **↓**BrdU/GFAP at 30 days post-ischemia	Leker et al. (2007)
C56BL/6J mice	8–10 weeks; Male	Induced MCAO Induced MCAO followed by ABAH (Myeloperoxidase inhibitor) treatment	N/S	**ABAH treatment:** ↑ BrdU/DCX, BDNF, p-CREB, AcH3, CXCR4 **↑** BrdU/Nestin in ipsilateral striatum	Kim et al. (2016)
Wistar rats	7–9 weeks; male	Induction of Cortical Spreading Depression (CSD group CSD+two vein occlusion (2VO) group, Sham group	**CSD + 2VO group**: ↑ BrdU	**CSD + 2VO group**: ↑ BrdU/DCX in ipsilateral and contralateral cortex **↑** BrdU/NeuN	Tamaki et al. (2017)
Sprague–Dawley rats	8–9 weeks; male	Induction of Spreading Depression (SD), 7 + 1 episode/hour	N/S	**↑**Vimentin day 3–6 in SPZ **↑** B-tubulinIII/BrdU – generated from nestin(+)/vimentin(−) cells	Xue et al. (2009)

#### Cortical-generated Neural Progenitors and Cortical Neurogenesis

Notably, the SVZ may not be the only supply of new cortical neurons following stroke, and NSCs/progenitor cells may reside in the cortex itself. Nakagomi et al. (2009) induced cerebral infarcts in 6-week-old mice and found nestin-positive cells 7 days post-stroke in the ipsilateral cortex. These nestin-positive cells co-labeled with stem cell markers S100 calcium-binding protein B (S100-B) and NG2 (the latter to a lesser extent) and produced neurospheres *in vitro* within 2 days of infarct. Neurosphere cells expressed Paired Box 6 (Pax6, a transcription factor implicated in the regulation of neurogenesis) and GFP, indicating that this vast network of neuronal precursors was limited to and expanded throughout the stroke area and that none had originated in the SVZ (Nakagomi et al., 2009). Similar findings were reported in a later study, which examined autoptic human cerebral cortex after cardiogenic embolism. Nestin-positive cells were abundant in the post-stroke cortex as well as Musashi-1-positive cells (another NSPC marker), although to a lesser extent. However, these stem cells were both β-tubulin III- and GFAP-negative, and therefore their potential to repair the injured cortex is uncertain (Nakayama et al., 2010).

While most of the abovementioned studies identified an endogenous pool of neuronal precursors within the cortex, none characterized the location of these precursors (Arsenijevic et al., 2001; Kornack and Rakic, 2001; Bernier et al., 2002). On the other hand, based on its unique morphology and expression of reelin (a regulator of neuronal migration), the neurogenic potential of cortical layer 1 has been an area of interest since the 1990s. Zecevic and Rakic (2001) first described the importance of this cortical layer in the developing primate brain. BrdU labeling *in utero* showed the development of large Cajal–Retzius cells that were positive for reelin, followed by a peak in the expression of GABAergic neurons (Zecevic and Rakic, 2001). However, in normal mice, while BrdU-labeled cells can be detected both in the cortex and the SVZ, the SVZ seems to be the major contributor for the generation of new cortical neurons. Furthermore, BrdU-positive cells in layer 1 only persisted for a limited time postnatally and were not present in adulthood. Furthermore, although 40% of layer 1 cells expressed NG2 (while SVZ cells did not express this marker), SVZ cells produced a greater proportion of pure neuronal cells (positive for both GFP and TuJ1). Layer 1 cells also did not express CR or reelin but were positive for GABA (Costa et al., 2007). Notably, transgenic mice (Pax-negative) showed increased proliferation and migration of BrdU-positive cells from layer 1 (Costa et al., 2007). Despite these results, layer 1 neurogenesis may occur following ischemia as indicated by the presence of phosphorylated histone H3- (pH3, a proliferation marker), Ki-67-, and BrdU-positive cells in this region. The majority of Ki-67-positive cells co-labeled with GFP (which was specifically targeted to incorporate into proliferating cells) and glutamic acid decarboxylase 67 (GAD-67, a protein involved in the synthesis of GABA). These triple-positive cells were largely localized in layer 1, and after the ischemic insult, an increased number of cells positive only for GAD-67 and GFP was found in layers 2–6, suggesting the migration of post-mitotic cells generated in layer 1 (Ohira et al., 2010). These cells were morphologically similar to neurons and successfully integrated into the existing neuronal circuitry; however, most c-Fos (an immediate early gene) expression disappeared after 4 weeks, suggesting that this was only a transient increase in the number of new neurons (Ohira et al., 2010).

#### Modulation of Cortical Neurogenesis

Various endogenous factors have been shown to modulate cortical neurogenesis. In the adult rat brain, Bcl-2 overexpression stimulates the production of newborn immature (TuJ1-positive) and mature (MAP-2-positive) neurons while also inhibiting the apoptosis of these newly generated neurons (Zhang R. et al., 2006). In addition, several studies have illustrated the importance of growth factors in cortical neurogenesis following ischemic insults. For instance, Kolb et al. (2007) showed that the combination of EGF with erythropoietin is able to stimulate the migration of cells from the SVZ to the damaged cortex, leading to tissue and functional recovery in adult male rats following stroke (Kolb et al., 2007). Similarly, transgenic mice overexpressing VEGF show an increased number of BrdU-positive cells in the SVZ after ischemic injury as well as increased neuroblast migration to the injured cortex, reduction in infarct size, and improved functional recovery (Wang et al., 2007). Furthermore, FGF-2 treatment was shown to result in long-term (up to 90 days) and sustained proliferation of BrdU-positive cells in rats following stroke. A subset of these cells stained positive for NeuN, and this subset increased with time (Leker et al., 2007). However, while ischemic conditions can stimulate neurogenesis, inflammatory factors such as myeloperoxidase can suppress this response to some extent. Indeed, Kim et al. (2016) demonstrated that myeloperoxidase inhibition with 4-aminobenzoic acid hydrazide resulted in an increased number of BrdU-labeled cells in the cortex and the striatum of male mice following stroke (Kim et al., 2016).

Various exogenous manipulations can also influence cortical neurogenesis in response to stroke/ischemia. Cortical spreading depression is a constant wave of depolarization that can be experimentally created through treatment of neurons with KCl (Tamaki et al., 2017). This technique can enhance cortical neurogenesis following ischemia by obstruction of two superficial cortical veins as indicated by BrdU and DCX labeling (Tamaki et al., 2017). These cells may be generated in cortical layer 1 as indicated by nestin expression and then migrate to other layers in the cortex (Xue et al., 2009). In addition, chronic stress induced by daily social defeat lowered the levels of neurogenesis in the rat cortex, and this deficit was shown to be partially restored by treatment with fluoxetine (Coe et al., 2003). On the other hand, a number of interventions known to induce neurogenesis in other parts of the brain do not have a neurogenic effect in the cortex. For example, environmental enrichment and physical exercise were shown to induce the production of new glial cells, but not neurons in the mouse cortex (Ehninger and Kempermann, 2003). In rats, electroconvulsive seizure treatment likewise produced new cortical cells that were positive for BrdU but failed to express neuronal markers, even after 4 weeks (Madsen et al., 2005).

### Neurogenesis in the Amygdala

#### Does Neurogenesis in the Amygdala Persist Into Adulthood?

A small body of evidence demonstrates neurogenic potential in the amygdala. Bernier et al. (2002) first demonstrated this phenomenon in monkeys and confirmed the neuronal lineage of newly generated cells by co-labeling with BrdU and either NeuN, MAP-2, or TuJ1. However, these authors hypothesized that the progenitor cells detected in this region actually originated in and migrated from the SVZ (Bernier et al., 2002). In a subsequent study, Saul et al. (2015) also demonstrated the presence of BrdU/DCX double-labeled cells in the adolescent rat amygdala; however, none of these immature neuroblasts went on to express markers of neuronal maturation 28 days after BrdU injection (Saul et al., 2015). Similarly, DCX labeling in macaque monkeys showed the presence of immature neurons in various regions of the amygdala; however, the neurogenic potential of this brain region appeared to diminish with age, in contrast to the findings reported in rats (Saul et al., 2014, 2015). Furthermore, a recent study has observed an endogenous population of precursor cells in the basolateral amygdala of mice that persists into adulthood (Jhaveri et al., 2018). This population was found to be significantly smaller than the ones observed in the hippocampus and SVZ but had the potential of generating neurospheres *in vitro* and to form mature interneurons as indicated by their electrophysiological properties (Jhaveri et al., 2018). However, it is possible that most of the new neurons generated in the amygdala originate from existing immature neurons with delayed maturation in the adult brain. A compelling study recently investigated this hypothesis and reported the existence of a population of cells expressing DCX + PSA−NCAM+ in the paralaminar nuclei of the human amygdala (Sorrells et al., 2019). These cells were found to be maintained in a state of protracted or arrested maturation and could be activated by genetic and environmental influences to functionally integrate into existing circuitries. Although the migration and the maturation of these neurons can happen at different ages, the authors observed that their most substantial contribution to the cytoarchitecture of the amygdala occurs during adolescence (Sorrells et al., 2019). Table 4 summarizes the studies that have evaluated the neurogenic capacity of the amygdala.

**Table 4:** Summary of studies investigating neurogenesis in the amygdala.

Species	Age/Sex	Manipulation/ Treatment	Proliferation	Survival/Differentiation	Reference
Squirrel Monkeys and *Cynomolgus* monkeys	3–12 years	1, 7, 14, 21 days after last injection	↑BrdU (at all time points)	GFAP, TuJ1, NeuN present in amygdala	Bernier et al. (2002)
Sprague–Dawley Rats	28–42 days; Male	1, 5, 10, 28, 56 days Stress	↑BrdU (highest at 24 h post-injection)	**↑**BrdU at all timepoints, DCX/BrdU at all timepoints, NG2/BrdU at all time points No NeuN/BrdU at any time point **Following stress response:** No difference in BrdU at 24 h ↓43% in BrdU at 10 days No difference in DCX/BrdU at 10 days ↓47% in NG2/BrdU	Saul et al. (2015)
*Rhesus* monkeys	12.1–31.3 years; Female and Male	Young adult, mid-age, and aged	-	DCX present in all age groups ↓41% mid-age ↓25% aged	Zhang et al. (2009)
C5BL/6J mice	7–112 weeks; Male	Contextual Fear conditioning	Ki-67 labeled cells in the Basolateral Amygdala (BLA)	**↑**DCX-GFP in BLA which exhibit electrophysiological properties of interneurons **↑**BrdU/NeuN	Jhaveri et al. (2018)
*Prairie voles*	85–135 days; Female	Isolation, female exposed, male exposed	BrdU in all treatment groups at 2 days (male exposure > isolation)	BrdU in all treatment groups at 3 weeks (male exposure > female exposure and isolation) TuJ1/BrdU present in amygdala, no difference between treatment groups	Fowler et al. (2002)
*Prairie voles* (*Microtus ochroasters*) Meadow voles (*M. Pennsylvanicus*)	4–5 months; Female	Species Estrogen Treatment	Meadow voles had higher BrdU compared to Prairie voles Following Estrogen treatment: ↑BrdU ↑ in BrdU was higher in meadow voles	**In pCorA and pMeA:** 40.5% TuJ1/BrdU 45.5% NG2/BrdU 14.0% BrdU No differences between treatment groups or species	Fowler et al. (2005)
Meadow voles (*M. Pennsylvanicus*)	2–3 months; Male	Oil vehicle, TP, DHT, estradiol benzoate	TP and EB treated: ↑ BrdU compared to oil treated and DHT (cortical and medial nuclei)	No differences between treatment groups: 44.2% TuJ1/BrdU 34.5% NG2/BrdU 21.3% BrdU Time course experiment: BrdU: 24 h>6 h = 1 h>30 min In MeA 24 h>30 min No differences in CorA or VMH	Fowler et al. (2003)
C57BL6/J mice CTR mice RUN mice	2 months; Female	Environment enrichment and physical activity 1 day vs. 4 weeks	-	NG2/BrdU 88.3% at 1 day; 79.6% at 4 weeks No NeuN/BrdU at 1 day or 4 weeks Of the NG2/BrdU cells: 95.6% S100ß at 1 day; 74.2% at 4 weeks Cells were also positive for DCX, NeuroD, BLBP, Olig2, vimentin, and Nestin, Iba Environmental enrichment and physical activity: ↓BrdU in ENR and RUN at 4 weeks ↓BrdU/Iba and ↓S100ß/BrdU in ENR and RUN groups at 4 weeks No difference in NG2/BrdU at 4 weeks	Ehninger et al. (2011)
C56BL/6 mice	8 weeks; Male	Standard, wide cages, enriched cages	No significant difference between BrdU at 0 weeks and 2 weeks	Enriched stimuli reduced BrdU-positive cell death: (standard: 30.9%; Wide: 27.6%; Enriched: 13.3%) No differences in numbers of BrdU cells, or cell differentiation between conditions After 3 weeks, most cells Olig-2/BrdU	Okuda et al. (2009)
C57BL/6 mice	2 months; Male	RMS lesioned	-	DCX/BrdU in amygdalopiriform area, amygdalohippocampal area, and multiple amygdaloid nuclei	Shapiro et al. (2009)
Wistar rats	8 weeks; Male	Sham vs. bulbectomized Water vs. Imipramine 3 weeks vs. 8 weeks	Scattered BrdU in amygdala	↑BrdU 3× by bulbectomy after 3 weeks 85% were DCX/BrdU ↑BrdU after 8 weeks Imipramine did not increase BrdU	Keilhoff et al. (2006)
Sprague Dawley rate	10 weeks; Female	Repeat seizures induced by Pentylenetetrazole	-	↑BrdU ↑NeuN	Park et al. (2006)
Human	Entire lifespan (post-mortem); Male and Female	-	Specific population in the paralaminar nuclei (arrested maturation): DCX+ PSA-NCAM+	Differentiation could be influenced by life experiences during adolescence/adulthood: T-box, brain 1 (TBR1) + VGLUT2+	Sorrells et al. (2019)

#### Modulation of Neurogenesis in the Amygdala

Hormones, various environmental factors, stress, brain lesions, and seizures have all been shown to trigger the generation of new neurons in the amygdala (Fowler et al., 2002; Keilhoff et al., 2006; Park et al., 2006; Okuda et al., 2009; Shapiro et al., 2009; Ehninger et al., 2011; Saul et al., 2015). A series of studies, using prairie voles, by Fowler et al. was the first to shed light on the modulation of amygdala neurogenesis (Fowler et al., 2002, 2003, 2005). For example, these authors demonstrated that, contrary to social isolation, male exposure was shown to significantly increase the number of BrdU-labeled cells in the amygdala of females and that these newly generated cells co-expressed TuJ1 and persisted beyond 3 weeks (Fowler et al., 2002). Moreover, treatment with estradiol benzoate increased the density of BrdU-labeled cells in certain regions of the meadow vole amygdala, and approximately 40% of these cells expressed TuJ1. Notably, this effect was not observed in prairie vole. Related to this, meadow voles showed a higher level of ER-alpha expression, which may have contributed to this species-dependent difference in amygdala neurogenesis (Fowler et al., 2005). A similar experiment was performed on adult male meadow voles treated with testosterone. Testosterone propionate increased the number of BrdU-positive cells in the amygdala, and 44% of these cells expressed neuronal markers. Curiously, in males, there was no evidence of neurons migrating from the RMS into the amygdala, but these rather appeared to be exclusively produced and retained within the amygdala (Fowler et al., 2003).

Given the effect of environmental enrichment on neurogenesis in other regions of the brain, two studies have also examined the effects that this environmental manipulation might have in the amygdala of mice (Okuda et al., 2009; Ehninger et al., 2011). Both studies showed increased levels of proliferating cells (BrdU- or NG2-positive); however, neither study demonstrated signs of new neuronal production but, rather, an increase in glial differentiation (Okuda et al., 2009; Ehninger et al., 2011). Saul et al. (2015) has also examined the effect of environmental stress on amygdala neurogenesis using a 3-day protocol of repeated variable stress. This stress paradigm had variable effects on different populations of cells in the amygdala: BrdU-labeled and NG2/BrdU-labeled cells decreased, while DCX/BrdU-labeled cells were not affected by stress. Notably, these changes may have consequences on the developing amygdala (Saul et al., 2015), highlighting the role of exposure to early stressful events to the development and the maturation of this brain region. Notably, it has been proposed that a link between olfactory and limbic neurogenesis lies in a fear conditioning response to a variety of stimuli, including olfactory stimuli (Shapiro et al., 2009). Interestingly, Keilhoff et al. demonstrated that bilateral olfactory bulbectomy actually increased the number of BrdU-positive cells in the basolateral amygdala, with the exact opposite effect seen in the hippocampus (Keilhoff et al., 2006). Similar findings have been reported after stress exposure, where a decrease in BDNF levels and dendritic complexity has been reported in the hippocampus in association with a simultaneous increase in the amygdala (Vyas et al., 2002; Lakshminarasimhan and Chattarji, 2012). Thus, it is likely that the new neurons observed in this region contribute to specific types of learning, particularly those related to fear conditioning and emotional memories (Hung et al., 2015). Another line of evidence shows that seizures induce significant cell death, followed by a subsequent increase in neurogenesis in the amygdala of rats (Park et al., 2006). These studies together clearly illustrate that the limited neurogenic capacity of the amygdala can be modulated by various intrinsic and extrinsic factors, similarly to what is observed in other neurogenic regions of the brain.

## Therapeutic Relevance of Adult Neurogenesis

While it is now well established that neurogenesis persists into adulthood in various regions of the brain, the physiological relevance of newly generated neurons in various brain regions as well as the extent and the significance of neurogenesis in the adult human brain are still not fully elucidated (Bergmann et al., 2012; Jessberger and Gage, 2014). The first evidence of neurogenesis in the adult human brain was reported in 1998 when BrdU-labeled cells were detected in the hippocampus of cancer patients (Eriksson et al., 1998). Ethical considerations prevent the replication of such a study, but recent advances in methodology have provided alternatives. C^14^ labeling in post-mortem samples have shown that 700 new neurons are added to the human hippocampus daily and that a decline in hippocampal neurogenesis occurs with age (Spalding et al., 2013). In agreement, in a subsequent study, a normal decrease in mRNA levels of neuronal proliferation markers (Ki-67) with age was also reported, and this reduction in proliferation may be associated with an age-related decline in cognition (Mathews et al., 2017). Conversely, a recent study showed that new neurons (DCX–PSA-NCAM-positive) in the granule cell layer of the hippocampus decline sharply in childhood and exist very scarcely in adulthood (Sorrells et al., 2018). However, an autopsy study on healthy individuals aged 14–79 showed that neural progenitors and immature neurons persist into late adulthood. Curiously, these data also suggested that a decline in neurogenesis may occur only in the context of neurologic disease (Boldrini et al., 2018). The continued progression of research methods is crucial to this field, with the use of positron emission tomography imaging presenting one possibility to further clarify the extent of neurogenesis in the human adult brain (Tamura et al., 2016). Nevertheless, the prospect of new neuron generation in the human brain presents an exciting area of inquiry relevant to chronic neurodegenerative disorders, acute neurologic conditions, and metabolic diseases as well as the treatment of these pathologies.

For example, using stem-cell-based therapies to treat or mitigate the effects of stroke may have tremendous clinical implications, with possible modalities including stem cell transplantation, manipulation of endogenous progenitor cells, and targeting of growth, migration, and differentiation factors. Indeed several possibilities for stem cell transplantation have already been described (Marlier et al., 2015). NSC transplantation in ischemic rat brains was shown to lead to functional recovery (Chu et al., 2005; Jiang et al., 2006; Darsalia et al., 2011; Song et al., 2011; Smith et al., 2012). Additionally, transplanted NSCs were shown to enhance the recovery process through the release of growth factors including VEGF and FGF-2 (Sun et al., 2003; Türeyen et al., 2005; Drago et al., 2013) and the downregulation of inflammatory factors such as interferon-γ, TNF-α, and interleukins (Bacigaluppi et al., 2009; Oki et al., 2012). Induced pluripotent stem cells (iPSCs, stem cells that are differentiated *in vitro* prior to implantation) have also been shown to reduce the area of damage due to infarct (Jiang et al., 2011; Yuan et al., 2013). However, mesenchymal stem cells (MSCs) present the most feasible possibility for cell grafting into infarcted brain tissue (Marlier et al., 2015). MSCs can be acquired from multiple sources, and similarly to NSCs, their implantation leads not only to the reduction of functional deficit (Kang et al., 2003; Horita et al., 2006; Gutiérrez-Fernández et al., 2011) but also to the release of numerous growth factors and cytokines that can enhance neurogenesis (Kurozumi et al., 2005; Bao et al., 2011). Indeed MSC transplantation in animals has shown continued success (Sarmah et al., 2017; Bedini et al., 2018; Tanaka et al., 2018) and has led to small trials in human stroke patients. These trials demonstrate that this is a safe therapeutic method with long-term benefits in function (Bang et al., 2005; Lee et al., 2010; Qiao et al., 2014). The bone marrow appears to be a viable source of mesenchymal stem cell transplantation in ischemic stroke patients (Suárez-Monteagudo et al., 2009; Bhasin et al., 2011; Moniche et al., 2015, 2016; Steinberg et al., 2016). Furthermore, it is believed that the exosomes (vesicles containing factors that promote neurogenesis) released by these MSCs may harbor a great benefit to the recovery process and are currently under investigation (Zhang and Chopp, 2016; Chen and Chopp, 2018). Based on these results, further trials in stem cell transplantation and the utility of exosomes may be warranted to properly assess their true efficacy. Alternatively, structural and functional recovery in the cortex could come from harnessing the endogenous pools of stem cells either within the cortex (Bernier et al., 2002; Dayer et al., 2005; Shapiro et al., 2009) or from another neurogenic brain region such as the SVZ (Arvidsson et al., 2002). In fact, neurogenesis in the cortex appears to be upregulated in response to ischemic stroke (Magavi et al., 2000; Jiang et al., 2001; Jin et al., 2006; Yang et al., 2007; Ohira et al., 2010). Similarly, the SVZ also appears to respond to cortical stroke with an upregulation of cell proliferation, which is then followed by the migration and the integration of new neurons into the damaged region of the cortex (Jin et al., 2001; Zhang et al., 2001, 2004; Bernier et al., 2002; Tonchev et al., 2005; Yamashita et al., 2006). Pharmacologic manipulation of molecular signals could serve to enhance this neuroprotective response and improve recovery (Marlier et al., 2015). Potential signals could include growth factors (e.g., IGF-1; Nishijima et al., 2010), neurovascular regulators (e.g., VEGF, angiopoietin-1, FGF-2; Jin et al., 2002; Shen et al., 2004; Wang et al., 2007), cytokines and chemokines (Goldberg and Hirschi, 2009; Yenari et al., 2010; Lin et al., 2015), and factors involved in the migration of stem cells from the SVZ (Arvidsson et al., 2002; Yamashita et al., 2006; Le Magueresse et al., 2012).

The effect of adult neurogenesis on neurodegenerative diseases is less certain and appears to be dependent on the neurogenic niche and timing during the natural course of the neuropathology. PD is a disease characterized by the chronic degeneration of dopaminergic neurons in the SN, leading to progressive decline in motor function (Lamm et al., 2014). Furthermore, several studies have shown a decrease in the generation of new neurons in the SN with PD, which may contribute to the pathophysiology of this neurodegenerative disease (Höglinger et al., 2004; Freundlieb et al., 2006; L’Episcopo et al., 2012). As such, strategies capable of promoting the generation of new dopaminergic neurons [i.e., TH-, DAT- (Kim et al., 2011), or DARPP-32-positive (Inta et al., 2015)] that can replace the degenerating neurons of the nigrostriatal pathway may provide valuable therapeutic options for this motor disorder (Lamm et al., 2014). Transforming growth factor alpha (TGF-α) has consistently been shown to increase new neuronal production in the striatum of PD models (Cooper and Isacson, 2004; de Chevigny et al., 2008); however, very few studies have shown differentiation into dopaminergic neurons (Kim et al., 2011). Comparable results have also been obtained by stimulation with FGF-2 (Peng J. et al., 2008), EGF (Winner et al., 2008), LGF (Gonzalo-Gobernado et al., 2009), PDGF, and BDNF (Mohapel et al., 2005). Treatment with dopamine receptor agonists is another alternative that has shown mixed results (Winner et al., 2009), with 7-hydroxy-N, N-di-n-propyl-2-aminotetralin, in particular, showing an increase in the number of TH-expressing neurons in the striatum, an effect that was accompanied by functional recovery (Van Kampen and Eckman, 2006). Notably, exercise may also increase neurogenesis in PD as demonstrated in several animal models and thus lead to improvement in motor function (Fisher et al., 2004; Smith et al., 2011). Future studies may investigate cell transplantation of *in*-*vitro*-generated dopaminergic neurons under the influence of growth factors (Kim et al., 2011) as well as different molecular signals that can induce new dopaminergic neuronal production *in vivo*. MSC-induced neurogenesis in PD is also under investigation in animal models. MSCs have shown the ability to promote SN neurogenesis while also displaying neuroprotective, angiogenic, and immunomodulatory benefits (Gugliandolo et al., 2017). MSCs can be derived from the bone marrow (Shetty et al., 2009; Danielyan et al., 2011; Park et al., 2012), umbilical cord (Xiong et al., 2011; Yan et al., 2013), and adipose tissue (Choi et al., 2015; Schwerk et al., 2015a,b) and potentially give rise to dopaminergic neurons. Continued success in animal models may warrant human trials in the future, particularly given the success of this therapy in stroke models.

HD primarily involves the degeneration of medium-sized GABAergic neurons in the striatum, with other brain regions (including the hippocampus) being affected later in the course of disease progression. Interestingly, however, a decrease in hippocampal neurogenesis (Gil et al., 2005; Simpson et al., 2011), but an increase in SVZ cell proliferation and subsequent migration to the striatum, has been reported in HD (Tattersfield et al., 2004; Batista et al., 2006). This increase in SVZ neurogenesis may represent an endogenous compensatory mechanism, which can, in turn, provide an avenue for slowing disease progression. BDNF is an important factor in striatal neuron differentiation and maturation, and BDNF signaling is known to be impaired in the HD brain (Altar et al., 1997; Zuccato et al., 2001; Ma et al., 2010). Notably, BDNF treatment increases SVZ neuronal production and recruitment of new neurons into the striatum (Cho et al., 2007). Furthermore, pharmacologic treatments (e.g., sertraline) may increase BDNF levels in the striatum, leading to reduced striatal atrophy and improved motor function (Borrell-Pages et al., 2006; Conforti et al., 2008; Duan et al., 2008; Peng Q. et al., 2008; Simmons et al., 2009; Gill et al., 2017). Moreover, FGF-2 can also increase the recruitment of new neurons into the striatum (Jin et al., 2005). Pre-clinical and clinical trials have also assessed the potential of stem cell therapies for the treatment of HD (Kopyov et al., 1998; Bachoud-Lévi et al., 2000; Hauser et al., 2002), and promising results have been reported with NSCs (Reidling et al., 2018), MSCs (Snyder et al., 2010), iPSCs (Al-Gharaibeh et al., 2017), and embryonic stem cells (Aubry et al., 2008; Zimmermann et al., 2016). Therefore, both the pharmacologic manipulation of endogenous SVZ neurogenesis and stem cell transplantation-induced neurogenesis may present promising strategies for the treatment of HD, and future studies are therefore warranted to further test these therapeutic options.

Lastly, the close relationship between hypothalamic neurogenesis and metabolic function must be emphasized. Neurogenesis in the hypothalamus can be a response mechanism to a variety of metabolic stressors (Sousa-Ferreira et al., 2014). For example, HFD leads to an acute production of new anorexigenic (POMC-expressing) neurons, which in turn can prevent excessive fat storage (Kokoeva, 2005; Gouazé et al., 2013). In agreement, blocking the generation of these new neurons causes rapid weight gain in response to HFD (Pierce and Xu, 2010; Lee et al., 2012). On the other hand, obesity has been shown to inhibit neurogenesis, leading to reduced NPY and POMC neurons, and ultimately metabolic dysregulation (Pierce and Xu, 2010; Li et al., 2012; McNay et al., 2012; Gouazé et al., 2013). Interestingly, calorie restriction in the context of obesity can restore hypothalamic neurogenesis (McNay et al., 2012), and stimulation of neurogenesis with growth factors can also affect weight gain (Kokoeva, 2005). These studies together illustrate that the interaction between metabolism and hypothalamic neurogenesis may play an important role in obesity. This relationship might provide avenues for therapeutic weight management in the future.

## Conclusion

The discovery of functionally significant neurogenesis in the mammalian hypothalamus, cortex, striatum, SN, and amygdala has important implications not only with regards to the function of these brain regions but also in the context of neuropathological conditions that specifically affect these brain structures. As such, the continued development of non-invasive techniques to study the neurogenic potential of these brain regions in humans is paramount. In addition, pharmacologic and stem-cell-based strategies capable of promoting neurogenesis in brain regions such as the cortex, SN, and striatum will require further investigation. This will ascertain the true therapeutic potential of promoting neurogenesis following cortical stroke/ischemia and in the context of various neurodegenerative disorders such as PD and HD, with the ultimate goal of promoting functional recovery.

## Author Contributions

MJ drafted the initial version of the manuscript. LB conceived the figures and provided critical input. EW conceived the tables. AP and S-YY provided critical input. JG-M conceived the outline of the manuscript, provided critical input, and revised the initial version of the manuscript. All authors contributed to the article and approved the submitted version.

## Conflict of Interest

The authors declare that the research was conducted in the absence of any commercial or financial relationships that could be construed as a potential conflict of interest.
